# Dual-energy X-ray absorptiometry derived knee shape may provide a useful imaging biomarker for predicting total knee replacement: Findings from a study of 37,843 people in UK Biobank^☆^

**DOI:** 10.1016/j.ocarto.2024.100468

**Published:** 2024-04-09

**Authors:** Rhona A. Beynon, Fiona R. Saunders, Raja Ebsim, Monika Frysz, Benjamin G. Faber, Jennifer S. Gregory, Claudia Lindner, Aliya Sarmanova, Richard M. Aspden, Nicholas C. Harvey, Timothy Cootes, Jonathan H. Tobias

**Affiliations:** a University of Bristol, Musculoskeletal Research Unit, Bristol Medical School, Bristol, United Kingdom; b University of Aberdeen, Centre for Arthritis and Musculoskeletal Health, Aberdeen, United Kingdom; c The University of Manchester, Division of Informatics, Imaging & Data Sciences, Manchester, United Kingdom; d University of Bristol, Medical Research Council Integrative Epidemiology Unit, Bristol, United Kingdom; e University of Southampton, MRC Lifecourse Epidemiology Centre, Southampton, United Kingdom; f NIHR Southampton Biomedical Research Centre, University of Southampton and University Hospital Southampton NHS Foundation Trust, United Kingdom

**Keywords:** Knee shape, Osteoarthritis, Statistical shape modelling, Osteophytes

## Abstract

**Objective:**

We aimed to create an imaging biomarker for knee shape using knee dual-energy x-ray absorptiometry (DXA) scans and investigate its potential association with subsequent total knee replacement (TKR), independently of radiographic features of knee osteoarthritis and established risk factors.

**Methods:**

Using a 129-point statistical shape model, knee shape (expressed as a B-score) and minimum joint space width (mJSW) of the medial joint compartment (binarized as above or below the first quartile) were derived. Osteophytes were manually graded in a subset of images and an overall score was assigned. Cox proportional hazards models were used to examine the associations of B-score, mJSW and osteophyte score with TKR risk, adjusting for age, sex, height and weight.

**Results:**

The analysis included 37,843 individuals (mean age 63.7 years). In adjusted models, B-score was associated with TKR: each unit increase in B-score, reflecting one standard deviation from the mean healthy shape, corresponded to a hazard ratio (HR) of 2.25 (2.08, 2.43), while a lower mJSW had a HR of 2.28 (1.88, 2.77). Among the 6719 images scored for osteophytes, mJSW was replaced by osteophyte score in the most strongly predictive model for TKR. In ROC analyses, a model combining B-score, osteophyte score, and demographics outperformed a model including demographics alone (AUC ​= ​0.87 vs 0.73).

**Conclusions:**

Using statistical shape modelling, we derived a DXA-based imaging biomarker for knee shape that was associated with kOA progression. When combined with osteophytes and demographic data, this biomarker may help identify individuals at high risk of TKR, facilitating targeted interventions.

## 1 Introduction

Knee osteoarthritis (kOA) exhibits distinct radiological features, including osteophyte formation and joint space narrowing (JSN), with JSN largely affecting the medial joint compartment in primary kOA. The Kellgren-Lawrence grading system, which encompasses both these elements, serves as a useful tool for classifying kOA severity in epidemiological studies [1]. However, it has limitations in predicting clinical outcomes, showing weak associations with both pain and function [2,3]. This highlights the need to consider whether other parameters could provide additional predictive information beyond established radiographic traits, with knee shape emerging as a potential influential factor. Notably, kOA is already recognised to be associated with varus and valgus malalignment, which can both result from and contribute to the disease by altering joint mechanics and increasing biomechanical stress [[4], [5], [6]].

Statistical shape modelling, facilitated by machine learning techniques, shows promise in identifying joint shape features associated with adverse clinical outcomes. However, its application in knee-related studies remains relatively unexplored, with current research primarily focused on three-dimensional (3D) imaging modalities [[7], [8], [9]]. For instance, Bowes et al. introduced the B-score [9], derived from a statistical shape model (SSM) applied to knee MRI images. The B-score, which specifically captured the shape of the femur bone, was associated with risk of current and future pain, functional limitation and total knee replacement (TKR), and its predictive accuracy was comparable to that of the Kellgren-Lawrence grade.

DXA imaging is gaining interest for joint shape evaluation due to its advantages of low radiation exposure and cost, and widespread availability. Moreover, modern high-resolution DXA scanners produce images of comparable quality to radiographs, making it a viable option for screening individuals at high risk of osteoarthritis progression. Notably, in the hip, shape variations seen on DXA, including reduced acetabular coverage and cam morphology, have already been linked to advanced disease [10,11], while statistical shape modes have identified changes in hip shape that are linked to disease progression [12].

In this study, our primary objectives were twofold: firstly, to evaluate the feasibility of measuring knee shape using DXA scans, and secondly, to investigate whether DXA-derived knee shape is associated with kOA progression, independently of other radiographic features of kOA. To achieve this, we developed and applied a statistical shape model (SSM) to approximately 40,000 knee DXA scans from the UK Biobank (UKB). We then examined the relationships between the top ten knee shape modes (KSMs) and risk of subsequent TKR. Following this, we derived a B-score by integrating TKR-associated changes across all KSMs. Finally, we investigated whether the relationship between knee shape and TKR risk, as reflected by B-score, was independent of radiographic features of kOA, by examining multivariable models which also included minimum joint space width (mJSW) and osteophyte classification (available in a subset of approximately 7000 scans).

## 2 Materials and methods

### 2.1 Participants

We used data from the UKB extended imaging study [13], a large-scale research study launched in 2014 with the aim of collecting medical imaging data, including DXA scans, from approximately 100,000 participants in the UKB [14]. The UKB recruited individuals aged between 40 and 69, with the baseline data collection phase spanning from 2006 to 2010. Participants underwent a thorough assessment at both baseline and imaging visits, which involved the completion of touch-screen questionnaires, nurse-lead interviews, and physical measurements. Further information was obtained via data linkage to electronic health records, including hospital episode statistics (HES) [15].

All subjects provided written informed consent before participation. UKB has full ethical approval from the National Information Governance Board for Health and Social Care and the North-West Multi-Centre Research Ethics Committee (11/NW/0382). Permission to access and analyse UKB data for this study was approved under UKB application number 17295.

### 2.2 Acquisition of knee DXA images

DXA scans were acquired using a Lunar iDXA scanner (GE Healthcare), with participants in a non-weight bearing supine position [16]. High-resolution DICOM format images were downloaded from the UKB showcase (downloaded in April 2021). Individuals with prior TKR were excluded from the analysis.

### 2.3 Ascertainment of outcomes

Our primary outcome of interest was TKR, with hospital-diagnosed knee osteoarthritis (HES-kOA), as a secondary outcome. Both were identified through linkage to the HES database, which uses codes from the International Classification of Diseases 9th (ICD-9) and 10th (ICD-10) revisions, as well as the Office of Population Censuses and Surveys (OPCS) Classification of Surgical Operations and Procedures, version 4 codes [17]. In this study, we identified cases of HES-kOA and TKR using the codes adopted from Zengini et al. [18] (Supplementary Table 1). All UKB participants were prospectively and retrospectively linked. Records were available from April 1st, 1997, and the data were downloaded in July 2023, capturing information up until the end of October 2022. For TKR, we were able to retrieve the date of surgery. However, for HES-kOA, we only have the date of its initial documentation in the medical records; hence, we are unable to ascertain the duration of the participant's condition, and the dataset is cross-sectional in nature.

### 2.4 Assessment of covariates

Participants’ height and weight were measured prior to imaging using standardised procedures [19]. Age and sex were collected at the time of enrolment into UKB and were self-reported.

### 2.5 Statistical shape modelling

SSM is a computational technique used in image analysis to quantify and analyse variations in the shape of objects. The methodologies employed in this study have been described previously [20,21]. Briefly, the technique involves placing points around each image within a set of training images. The points represent the shapes of the bones on each image. The shapes are aligned to a reference frame and Principal Component Analysis (PCA) is applied to identify and quantify independent modes of shape variation (here termed knee shape modes [KSMs]). Any shape can be represented concisely using a vector of the weights on each KSM. Once the SSM is constructed, it can be used to generate new shapes that fall within the observed statistical range in the original dataset.

In this study, SSM was performed using the BoneFinder® software developed at The University of Manchester [22]. A 129-point template outlined the distal femur, proximal tibia, proximal fibula, and superior patella, while excluding any osteophytes (Supplementary Fig. 1). An automated search model was trained on ∼7000 images. Within the training set, 20% of the images were randomly chosen from individuals with self-reported non-specific osteoarthritis to enhance the model's learning of OA patterns. The remaining 80% were randomly selected, ensuring an equal sex distribution, as described previously [10]. Cross-validation experiments showed the points could be found on a new image with a mean point-to-point error of <3 ​mm for 95% of the images (Supplementary Fig. 2). The BoneFinder® model, employing a random-forest-based algorithm, automated point placement on the remaining DXA images (n ​= ​31,207 after exclusions). Trained annotators (RB and FS) manually refined point placements for 4214 images, enhancing SSM precision (Supplementary Table 2). The final SSM model was built on 37,927 DXA images. The 27 KSMs derived from the SSM were standardized using their respective sample standard deviations.

### 2.6 Generation of a quantitative measure of knee shape: B-score

To integrate the information from all KSMs, we employed the methodology introduced by Bowes et al. [9] to generate an overall knee shape variable termed the B-score. Using all 27 KSMs from the SSM, we derived mean shapes for a “diseased group” (including knees with either TKR or HES-kOA, depending on the specific outcome) and a “healthy group” (comprising knees that did not advance to TKR or did not have HES-kOA). For each outcome, we constructed a vector connecting the mean shapes of the diseased and healthy groups. The KSMs for each image were then orthogonally projected onto this vector. The SD of the projections from healthy cases was calculated and used to normalize all projections, resulting in the generation of the B-scores. Each increment in the B-score represents one SD from the mean knee joint shape of the healthy population (assigned a B-score of 0). As a sensitivity analysis, we calculated sex-specific B-scores. Separate sets of knee shape data were considered for each sex, and the calculations followed the same steps as described above for the overall B-score. Specifically, for each sex, we calculated the direction vectors based on the difference between the mean cases and the control shapes specific to that sex and divided the projections of knee shapes by the sex-specific standard deviations obtained from the healthy group.

### 2.7 DXA-based measures of joint space and osteophytes

A custom script automatically measured mJSW of the medial and lateral compartments using specific template points on the distal femur and proximal tibia (distal femur: medial points 24–30, lateral points 15–20; proximal tibia: medial points 68–73, lateral points 57–62; see Supplementary Fig. 1). We divided the mJSW in each compartment into quartiles and generated a binary variable that denoted whether the mJSW fell within the first quartile (corresponding to the smallest mJSW value) on the medial side.

Osteophytes were assessed in the sub-sample of 6719 DXA images used in the search training set. Each image was visually assessed for osteophytes on a 0–3 scale, referencing a DXA-based atlas created by RB and FS (supplementary doc) with input from DW (see acknowledgements). Intra-observer repeatability was assessed on a random sample of 200 images, demonstrating good agreement (κ ​= ​0.80). Cumulative values (ranging from 0 to 12) were computed by aggregating grades from all four sites on the medial and lateral aspects of the femur and tibia. An osteophyte score was then assigned based on the total sum: 0 (sum ​= ​0), 1 (sum ​= ​1), 2 (sum ​= ​2–3), 3 (sum ​= ​4 or greater), as outlined in Supplementary Table 3.

### 2.8 Statistical analysis

To assess the comparative strength of the relationships between KSMs, B-scores, mJSW, and osteophytes with the incidence of TKR and HES-kOA, we employed Cox proportional hazards modelling and logistic regression, respectively. The proportional hazards assumption was verified using the Schoenfeld residuals approach. Results are reported as hazard ratios (HRs) and odds ratios (ORs) with their corresponding 95% confidence intervals (CIs). Both unadjusted and adjusted analyses were conducted, with adjustments made for age, sex, height, and weight. Height and weight were chosen rather than BMI, which is a linear combination of the two, because individuals with the same BMI may have different body compositions, which may in turn contribute differently to biomechanical forces. Age, height, and weight were treated as continuous variables, while sex was considered as a binary variable. Where death occurred before TKR, the event was censored at the time of death.

We next constructed multivariable models to investigate whether relationships between knee shape, as reflected by B-score, and our outcomes were independent of mJSW and osteophytes. We began by fitting unadjusted models for B-score, mJSW, and osteophyte score in the subset of participants with osteophyte data (n ​= ​6719). We then introduced demographic factors (age, sex, height, and weight). Subsequently, we assessed models with an additional variable, which could be either B-score, mJSW, or osteophyte score, depending on the specific model. Finally, we examined a comprehensive model that included all DXA-derived variables and demographic factors. Goodness of fit was evaluated using Akaike information criterion (AIC) and Bayesian information criterion (BIC), while discriminative ability was assessed using either the area under the receiver operating characteristic curve (AUC) or the Harrell's Concordance index (C-index) index, for logistic and Cox regression models, respectively.

To further evaluate the discriminative ability of the models, we plotted receiver operating characteristic (ROC) curves for HES-kOA and TKR at 5 years. Chi-squared tests were employed to examine the equality of the area under the curves.

Analyses were conducted using Stata version 17 (StataCorp. 2021. *Stata Statistical Software: Release 17*. College Station, TX: StataCorp LLC.) and python 3.10.5.

## 3 Results

### 3.1 Participant characteristics

**Fig. 1:**
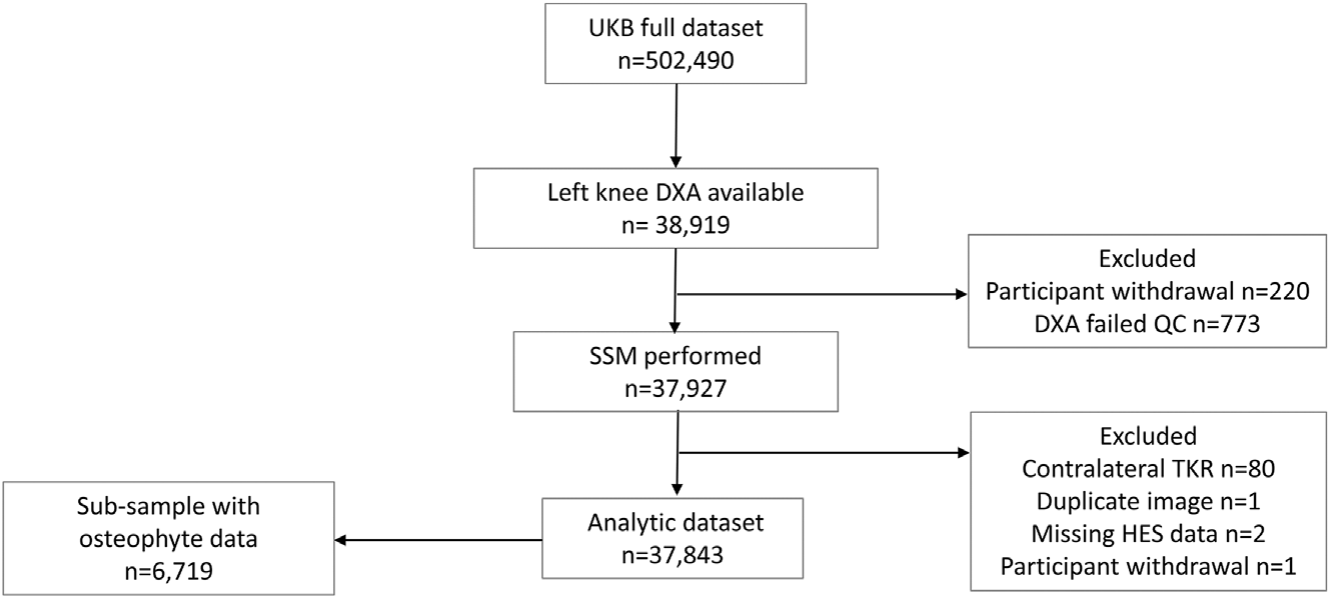
Flow Diagram of Participant Progression through the Study. At the time of the analysis, approximately 39,000 left knee DXA scans were available. DXA images underwent a comprehensive assessment to determine their suitability for inclusion in the SSM. Reasons for exclusion included: poor image quality, artefacts, positioning issues, short femoral or tibial shafts, and search failure. A total of 220 participants withdrew from the study, and an additional 80 participants were excluded due to having undergone TKR on the contralateral knee before obtaining the DXA image of the left knee. All participants in the analytic dataset had SSM data available, which was used to derive B-score and minimum joint space width (mJSW). Within this dataset, a sub-sample of 6719 participants had additional osteophyte data available. This sub-sample, comprising participants with B-scores, mJSW and osteophyte data, were used to develop an imaging biomarker for predicting TKR. Abbreviations: QC, quality control; SSM, statistical shape model; TKR, total knee replacement; UKB, UK Biobank.

In total 37,843 participants had SSM and clinical data available (Fig. 1). A total of 477 participants underwent TKR (1.26%; Table 1), with an average time to surgery of 2.81 years (SD ​= ​1.91 years; males ​= ​2.90 years [1.94], females ​= ​2.72 years [1.90]). There were 704 deaths during the study period. The sub-sample with osteophyte data (n ​= ​6719) were comparable to the full dataset with respect to their baseline demographics (Table 1), but the proportion of individuals with TKR was higher (2.19%, n ​= ​147). The average time to TKR in this sub-sample was 3.45 years [SD: 2.01; males: 3.81 [1.93], females: 3.14 [2.03]) and there were 185 deaths.

**Table 1:** Descriptive characteristics of the total study population and the subset in which osteophytes were assessed.

	Full dataset			Osteophyte dataset		
	Female	Male	Total	Female	Male	Total
	N ​= ​19710	N ​= ​18133	N ​= ​37843	N ​= ​3403	N ​= ​3316	N ​= ​6719
	*Mean (SD)*			*Mean (SD)*		
Age (years)	63.03 (7.41)	64.41 (7.64)	63.69 (7.55)	62.08 (7.25)	63.48 (7.65)	62.77 (7.48)
Height (cm)	163.64 (6.41)	177.23 (6.62)	170.15 (9.40)	163.36 (6.43)	176.90 (6.63)	170.04 (9.40)
Weight (kg)	68.11 (12.91)	83.08 (13.38)	75.29 (15.12)	68.79 (12.70)	83.65 (13.71)	76.12 (15.15)
mJSW medial compartment (mm)	3.63 (0.60)	4.28 (0.71)	3.94 (0.73)	3.55 (0.70)	4.24 (0.81)	3.89 (0.83)
mJSW lateral compartment (mm)	3.78 (0.83)	4.65 (0.84)	4.20 (0.94)	3.97 (0.94)	4.82 (0.94)	4.39 (1.03)
B-score knee replacement	−0.08 (0.99)	0.11 (1.02)	0.01 (1.01)	−0.03 (1.02)	0.08 (1.01)	0.02 (1.02)
B-score HES-kOA	−0.12 (1.00)	0.20 (1.02)	0.03 (1.02)	−0.04 (1.03)	0.13 (1.02)	0.04 (1.03)
	*N (%)*			*N (%)*		
HES-kOA						
no	18931 (96.05%)	17243 (95.09%)	36174 (95.59%)	3206 (94.21%)	3119 (94.06%)	6325 (94.14%)
yes	779 (3.95%)	890 (4.91%)	1669 (4.41%)	197 (5.79%)	197 (5.94%)	394 (5.86%)
Knee replacement						
no	19462 (98.74%)	17904 (98.74%)	37366 (98.74%)	3323 (97.65%)	3249 (97.98%)	6572 (97.81%)
yes	248 (1.26%)	229 (1.26%)	477 (1.26%)	80 (2.35%)	67 (2.02%)	147 (2.19%)
Osteophyte score						
grade 0-1				2423 (71.20%)	2456 (74.07%)	4879 (72.61%)
grade 2-3				980 (28.80%)	860 (25.93%)	1840 (27.39%)

The table presents the demographic and clinical characteristics of the study population, comprising all participants with statistical shape modelling data. The left-hand side provides information for the full dataset, while the right-hand side focuses on the sub-sample of participants who had additional osteophyte data available. Abbreviations: HES-kOA, hospital-diagnosed knee osteoarthritis; mJSW, minimum joint space width; SD, standard deviation; TKR, total knee replacement.

### 3.2 Relationship between knee shape and kOA outcomes

**Fig. 2:**
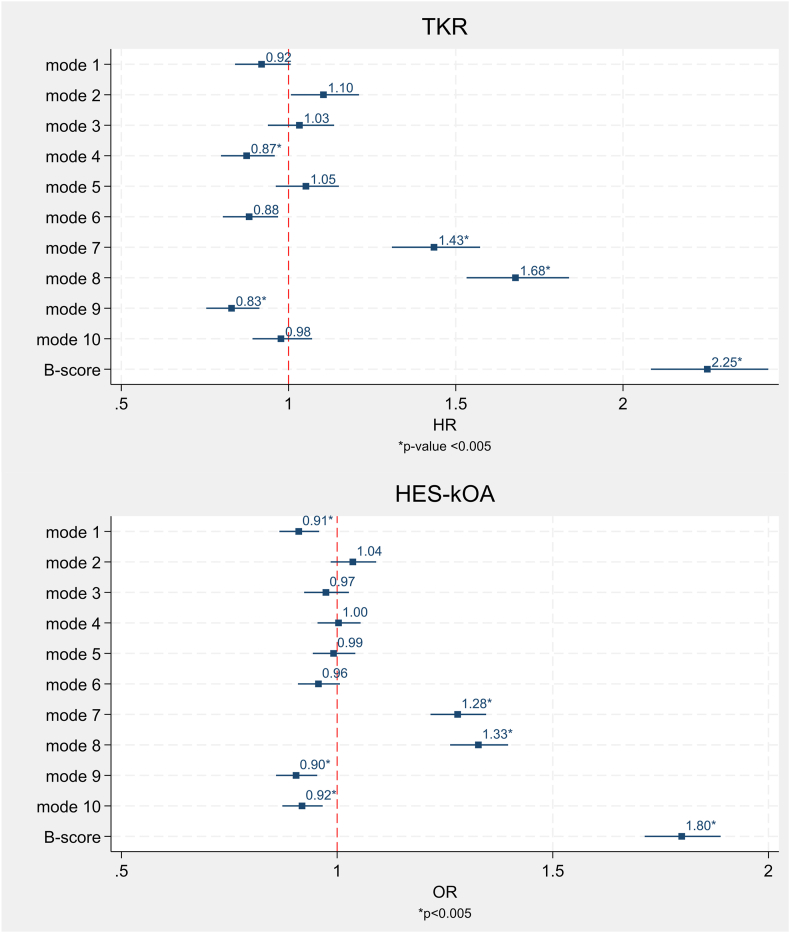
Associations between the top 10 knee shape modes and B-score with knee osteoarthritis outcomes (n ​= ​37,843). The top panel displays the association of knee shape with total knee replacement (TKR), whilst the bottom panel presents the association of knee shape with hospital diagnosed knee osteoarthritis (HES-kOA). Hazard ratios (HRs) and odds ratios (ORs) indicate the change in risk of TKR and HES-kOA per standard deviation increase in knee shape mode (KSM) and per standard deviation increase in B-score. Models are adjusted for age, sex, height and weight. 95% confidence intervals (CI) are provided. Associations that met the Bonferroni-significant threshold of p ​< ​0.005 are marked with an asterisk.

We initially evaluated individual modes of variation (KSMs) in relation to kOA outcomes. To limit multiple comparisons, we focused on the first 10 KSMs, explaining 79.5% of shape variance. Subsequent KSMs each made minimal contributions to the sample variance (≤2%) (Supplementary Fig. 3). Adjusted analysis results are presented graphically in Fig. 2, with unadjusted and adjusted associations detailed in Supplementary Table 4. A description of each KSM is provided in Supplementary Table 5.

Following adjustment for age, sex, height and weight, KSMs 4,7, 8, and 9 showed strong evidence of an association with TKR. Specifically, for each standard deviation (SD) increase in KSMs 4 & 9 there was a 13% and a 17% reduced hazard for TKR, while analogous increases in KSM7 and KSM8 were associated with a 43% and 68% increased hazard, respectively.

Five KSMs displayed strong statistical evidence of an association with HES-kOA in adjusted models. Increases in modes 1, 9, and 10 were linked to reduced odds of 9%, 10%, and 8%, whilst increases in modes 7 and 8 were associated with 28% and 33% increased odds, respectively.

Given that variations in knee shape could be spread over multiple modes, we next assessed the relationship between B-score, a single variable combining shape information across all KSMs, and kOA outcomes. B-score distribution is shown in Supplementary Fig. 4.

We observed strong positive associations between B-scores and our outcomes (Fig. 2; additional data in Supplementary Table 4). After adjustment, each incremental increase in B-score (which represents a SD from the mean healthy shape), corresponded to a 2.3-fold increased hazard for TKR. Similar results were found in the sex-stratified analysis (Supplementary Table 6). Additionally, each SD increase in B-score was associated with increased odds of HES-kOA, with a 1.8-fold higher likelihood. Stratifying by sex did not alter these findings.

**Fig. 3:**
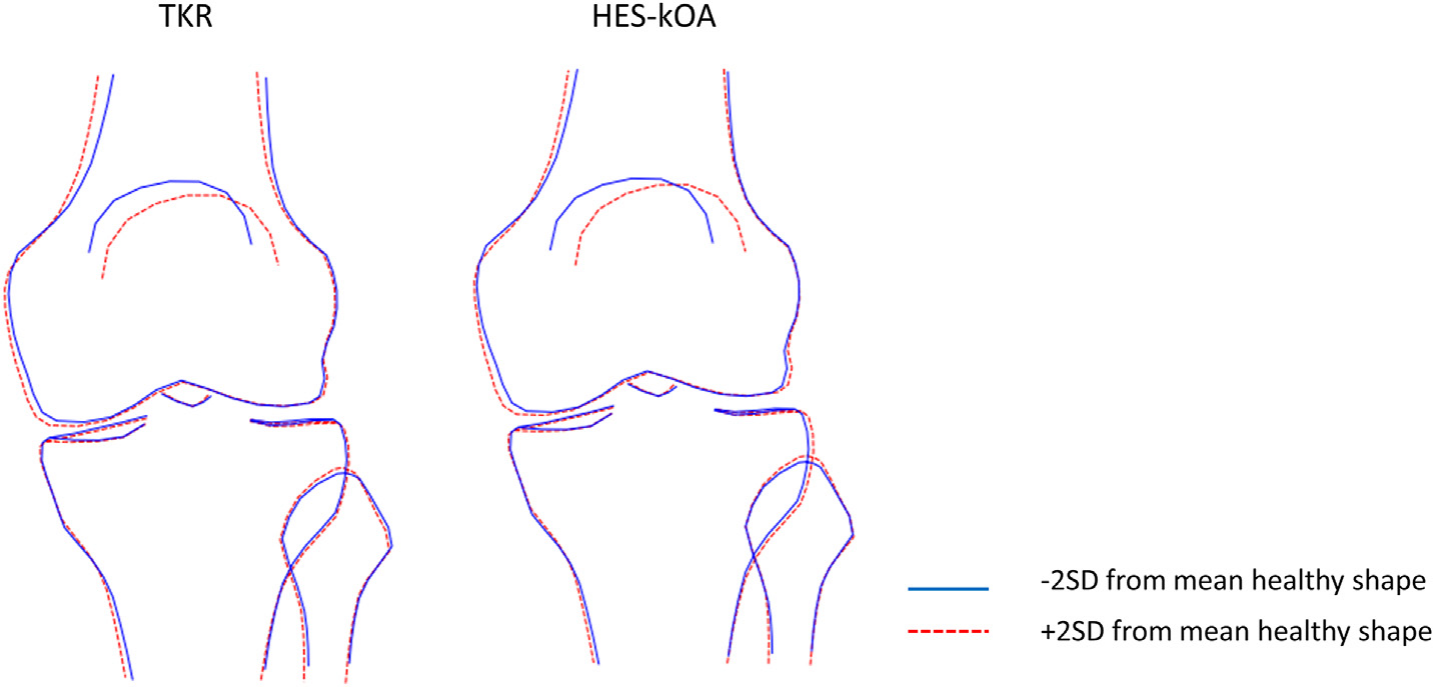
Examples of differences in bone shape corresponding to an increase and decrease in B-scores. B-scores were obtained by projecting all statistical knee shape modes (KSMs) onto a vector connecting healthy and diseased knee joint shapes. The diseased population included individuals who underwent TKR (left) or had hospital-diagnosed knee osteoarthritis (HES-kOA) (right). The figure illustrates shape changes associated with ±2 standard deviations (SD) from the mean B-score. The solid blue line depicts the shape at -2SD, while the dashed red line represents the shape at +2SD. (For interpretation of the references to color in this figure legend, the reader is referred to the Web version of this article).

Fig. 3 depicts joint shape variations associated with B-scores ±2SD from the healthy group mean. An increase in B-score is linked with increasing varus alignment accompanied by reduced medial joint space width, widening of the femoral articular surface, and a more lateral position of the patella relative to the inferior femur.

### 3.3 Relationship between mJSW and kOA outcomes

Results are presented in Table 2 (Supplementary Table 7 for unadjusted results). Individuals in the first quartile of medial mJSW, representing the narrowest mJSW, had a 2.5-fold increased hazard for TKR and 53% higher odds of HES-kOA, compared with those in the fourth quartile. Conversely, when examining lateral mJSW, the first and second quartiles showed a lower risk of TKR when compared with the fourth.

**Table 2:** Associations of minimum joint space width (mJSW) with HES-kOA and TKR (n ​= ​37,843).

	TKR				HES-kOA			
	*HR*	*ll*	*ul*	*p-value*	*OR*	*ll*	*ul*	*p-value*
*Medial compartment*								
1st quartile	2.54	1.92	3.37	**7.29 x 10**^−^**^11^**	1.53	1.32	1.78	**2.70 x 10**^−^**^08^**
2nd quartile	1.32	0.97	1.79	0.074	1.10	0.94	1.28	0.222
3rd quartile	0.96	0.70	1.32	0.823	0.96	0.83	1.12	0.599
*Lateral compartment*								
1st quartile	0.77	0.58	1.01	0.058	0.83	0.71	0.97	**1.70 x 10**^−^**^02^**
2nd quartile	0.60	0.45	0.79	**3.18 x 10**^−^**^04^**	0.72	0.62	0.84	**1.49 x 10**^−^**^05^**
3rd quartile	0.81	0.63	1.03	0.084	0.82	0.72	0.94	**0.004**
*Binary Q1 vs ​> ​Q1*	2.28	1.88	2.77	**<0.001**	1.49	1.33	1.67	**6.15 x 10**^−^^**12**^

When examining the association between quartiles of minimum joint space width (mJSW) and the risk of total knee replacement (TKR) and hospital diagnosed knee osteoarthritis (HES-kOA), we designated quartile 4 as the reference category (i.e., the greatest mJSW). Odds ratios (ORs) and hazards ratios (HRs) represent the difference in risk for someone in the first, second or third quartile versus the fourth quartile. For the binary mJSW variable, the HRs and ORs quantify the risk variation associated with values above the first quartile in the medial compartment, in comparison with the first quartile (representing the “unhealthiest” mJSW). CI, 95% confidence interval; Q1, first quartile of mJSW (medial compartment). Models were adjusted for age, sex, height and weight. p ​< ​0.05 are shown in bold.

Considering the binary mJSW variable, individuals in the first quartile on the medial side had a 2.3-fold higher hazard for TKR, compared with individuals in higher quartiles. They also experienced 49% increased odds of HES-kOA.

### 3.4 Relationship between osteophytes and kOA outcomes

The prevalence of manually graded osteophytes (n ​= ​6719) are provided in Supplementary Table 8. The regression analysis (Table 3, with unadjusted results in Supplementary Table 9) revealed a consistent pattern of increasing HRs and ORs for kOA outcomes with higher osteophyte grades. This was apparent across all anatomical sites.

**Table 3:** Association of osteophyte grades with TKR and HES-kOA (n ​= ​6719).

	TKR				HES-kOA			
	*HR*	*ll*	*ul*	*p-value*	*OR*	*ll*	*ul*	*p-value*
**Medial femur**								
grade 1	2.58	1.69	3.93	1.11 x 10^−^^05^	1.56	1.21	2.00	0.001
grade 2	9.84	6.19	15.65	4.27 x 10^−^^22^	6.16	4.44	8.54	1.02 x 10^−^^27^
grade 3	20.95	12.88	34.06	1.40 x 10^−^^34^	9.21	5.93	14.30	4.87 x 10^−^^23^
**Lateral femur**								
grade 1	2.25	1.25	4.04	0.007	2.10	1.42	3.10	2.10 x 10^−^^04^
grade 2	7.61	4.59	12.62	3.40 x 10^−^^15^	6.66	4.46	9.96	2.28 x 10^−^^20^
grade 3	14.22	8.71	23.21	2.57 x 10^−^^26^	6.75	4.01	11.38	7.41 x 10^−^^13^
**Medial tibia**								
grade 1	3.60	2.47	5.24	2.39 x 10^−^^11^	3.08	2.46	3.85	7.60 x 10^−^^23^
grade 2	12.01	7.32	19.70	7.85 x 10^−^^23^	6.15	4.05	9.32	1.29 x 10^−^^17^
grade 3	22.88	11.31	46.28	3.08 x 10^−^^18^	14.95	6.91	32.33	6.25 x 10^−^^12^
**Lateral tibia**								
grade 1	3.00	2.05	4.39	1.36 x 10^−^^08^	2.00	1.61	2.50	7.17 x 10^−^^10^
grade 2	9.41	5.38	16.45	3.64 x 10^−^^15^	5.66	3.68	8.71	3.16 x 10^−^^15^
grade 3	31.56	16.85	59.09	4.00 x 10^−^^27^	13.78	6.91	27.46	9.03 x 10^−^^14^
**OP score**								
1	1.81	0.94	3.49	0.076	1.56	1.14	2.14	0.005
2	4.62	2.62	8.14	1.19 x 10^−^^07^	2.76	2.06	3.70	9.28 x 10^−^^12^
3	21.64	12.56	37.29	1.68 x10 ^−^^28^	9.78	7.16	13.36	<0.001

For individual osteophyte grades, hazard ratios (HRs) and odds ratios (ORs) represent the change in risk compared with grade 0 osteophytes. The osteophyte scores were determined based on the cumulative sum of the four individual osteophyte grades: 0 (sum ​= ​0), 1 (sum ​= ​1), 2 (sum ​= ​2–3), and 3 (sum ​= ​4 or greater). In analysing the association between osteophyte scores and the probability of total knee replacement (TK)R and hospital diagnosed knee osteoarthritis (HES-kOA), the reference category was established as 0. The HRs and ORs quantify the change in risk for individuals with scores of 1, 2, or 3 relative to those with a score of 0. Models were adjusted for age, sex, height and weight. CI, 95% confidence intervals; HES-kOA, hospital diagnosed knee osteoarthritis; TKR, total knee replacement. Abbreviations: CI, 95% confidence intervals.

An osteophyte score of 3 (derived from the sum of the osteophyte grades) was associated with a 22-fold increased hazard for TKR and 10-fold increased odds of HES-kOAK, compared to a score of 0.

### 3.5 Performance of multivariable models in predicting TKR

Table 4 presents a comparison of predictive performance among different models for TKR, incorporating B-score, binary mJSW, osteophyte score, and demographic variables within the sub-group of 6719 participants.

**Table 4:** Predictive performance of B-scores, binary mJSW and osteophyte score with TKR and HES-kOA (n ​= ​6719).

TKR							
Model	HR	ll	ul	*p*-value	AIC	BIC	C-stat
B-score	2.59	2.24	3.00	2.81 x 10^−^^37^	2390.78	2397.59	0.75
B-score ​+ ​demo	2.33	2.00	2.71	8.03 x 10^−^^28^	2329.45	2363.51	0.81
B-score ​+ ​demo ​+ ​mJSW	2.23	1.90	2.62	1.28 x 10^−^^22^	2329.16	2370.04	0.81
B-score ​+ ​demo ​+ ​OP	1.74	1.49	2.03	2.51 x 10^−^^12^	**2224.79**	**2265.66**	0.86
B-score ​+ ​demo ​+ ​mJSW ​+ ​OP	1.67	1.42	1.97	8.38 x 10^−^^10^	2225.13	2272.82	0.86
mJSW	2.68	1.94	3.70	2.50 x 10^−^^09^	2508.48	2515.29	0.61
mJSW ​+ ​demos	2.24	1.59	3.17	4.31 x 10^−^^06^	2422.51	2456.57	0.76
mJSW ​+ ​demos ​+ ​B-score	1.32	0.92	1.89	0.130	2329.16	2370.04	0.81
mJSW ​+ ​demos ​+ ​OP	1.86	1.33	2.61	<0.001	2261.53	2302.40	0.85
mJSW ​+ ​demo ​+ ​B-score ​+ ​OP	1.27	0.88	1.82	0.197	2225.13	2272.82	0.86
OP	3.50	2.93	4.18	<0.001	2314.17	2320.99	0.80
OP ​+ ​demo	3.04	2.53	3.64	4.76 x 10^−3^^3^	2272.29	2306.36	0.85
OP ​+ ​demo ​+ ​B-score	2.46	2.04	2.96	1.18 x 10^−^^21^	**2224.79**	**2265.66**	0.86
OP ​+ ​demo ​+ ​mJSW	2.94	2.45	3.52	1.11 x 10^−^^31^	2261.53	2302.40	0.85
OP ​+ ​demo ​+ ​B-score ​+ ​mJSW	2.45	2.04	2.95	1.48 x 10^−^^21^	2225.13	2272.82	0.86
HES-kOA							

**Model**	**OR**	**ll**	**ul**	***p*-value**	**AIC**	**BIC**	**AUC**
B-score	1.98	1.79	2.19	<0.001	2820.73	2834.36	0.68
B-score ​+ ​demo	1.85	1.67	2.05	3.63 x 10^−^^31^	2768.53	2809.41	0.71
B-score ​+ ​demo ​+ ​mJSW	1.82	1.64	2.03	2.05 x 10^−^^28^	2769.10	2816.79	0.71
B-score ​+ ​demo ​+ ​OP	1.57	1.41	1.74	5.53 x 10^−^^17^	**2641.17**	**2688.86**	0.76
B-score ​+ ​demo ​+ ​mJSW ​+ ​OP	1.55	1.39	1.72	2.62 x 10^−^^15^	2642.15	2696.65	0.76
mJSW	1.65	1.33	2.05	4.79 x 10^−^^06^	2983.58	2997.21	0.55
mJSW ​+ ​demo	1.54	1.22	1.94	2.47 x 10^−^^04^	2892.23	2933.11	0.65
mJSW ​+ ​demo ​+ ​B-score	1.16	0.91	1.47	0.229	2769.10	2816.79	0.71
mJSW ​+ ​demo ​+ ​OP	1.43	1.13	1.81	0.003	2704.43	2752.12	0.73
mJSW ​+ ​demo ​+ ​B-score ​+ ​OP	1.13	0.89	1.45	0.310	2642.15	2696.65	0.76
OP	2.25	2.04	2.50	<0.001	2748.39	2762.01	0.71
OP ​+ ​demo	2.08	1.87	2.30	<0.001	2711.18	2752.06	0.73
OP ​+ ​demo ​+ ​B-score	1.84	1.65	2.05	5.91 x 10^−^^29^	**2641.17**	**2688.86**	0.76
OP ​+ ​demo ​+ ​mJSW	2.06	1.85	2.28	<0.001	2704.43	2752.12	0.73
OP ​+ ​demo ​+ ​B-score ​+ ​mJSW	1.84	1.65	2.05	7.04 x 10^−^^29^	2642.15	2696.65	0.76

The table includes results of univariable and multivariable models (left), and measures of model fit and discrimination (right). Demographic characteristics include age, sex, height and weight. For B-score, HRs and ORs indicate the change in risk per standard deviation increase in B-score. For mJSW, HRs and ORs reflect the risk difference between the first quartile (on the medial compartment) compared with quartiles two, three, and four. Osteophyte score was entered into the model as a linear term and therefore HRs and ORs represent the difference in risk per unit increase in score. When evaluating the predictive accuracy of the time-to-event (cox) models we used Harrell's C-index. For logistic regression models, where the outcome was binary, we used the AUC. The most parsimonious model, containing the predictors B-score, osteophyte grade and demographic variables, is shown in bold.

Abbreviations: AIC, Akaike information criterion; AUC, area under the receiver operating characteristic curve; BIC, Bayesian information criterion; CI, 95% confidence intervals; C-index, Harrell's concordance index; Demo, demographic variables; OP, osteophyte score; HR, hazard ratio; mJSW, minimum joint space width; OR, odds ratio; TKR, total knee replacement.

In the unadjusted models, all DXA-derived variables exhibited associations with TKR, with the osteophyte score demonstrating the highest predictive capability. The inclusion of demographic variables attenuated these individual associations but improved overall predictive accuracy.

In the multivariable models, incorporating demographic variables and mJSW led to a modest reduction in the association between B-score and TKR, with the HR decreasing from 2.59 (95% CI: 2.24, 3.00) to 2.23 (95% CI: 1.90, 2.62). However, upon inclusion of demographic variables and osteophyte score, the association considerably weakened, experiencing a reduction in strength of approximately 50% (HR ​= ​1.74 [95% CI: 1.49, 2.03]). This model also demonstrated the best overall fit, indicated by the lowest Akaike Information Criterion (AIC) and Bayesian Information Criterion (BIC), and the highest discrimination, as indicated by the C-index.

**Fig. 4:**
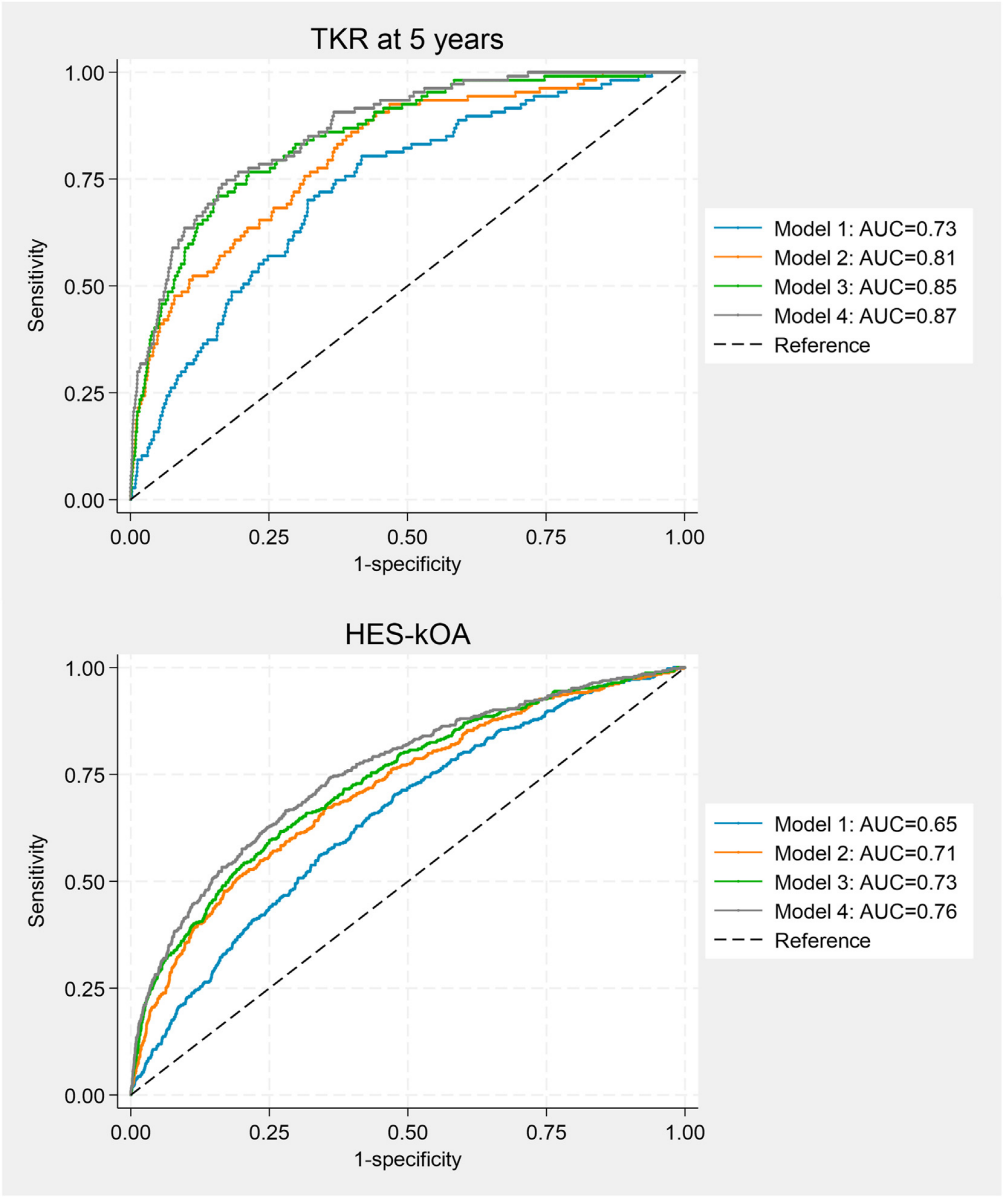
Receiver Operating Characteristic (ROC) Curve for Prediction of HES-kOA and TKR at 5 ​Years (n ​= ​6719). Model 1: age, sex, height, and weight; Model 2: age, sex, height, weight, B-score; Model 3: age, sex, height, weight, binary osteophyte grade; Model 4: age, sex, height, weight, B-score, binary osteophyte grade. Abbreviation: AUC, area under the receiver operating characteristic curve.

Subsequently, we examined the predictive ability of this model in classifying TKR at five years. Demographic factors alone achieved an AUC of 0.73 (Fig. 4), while the optimal model (including osteophyte score, B-score, and demographics) had an AUC of 0.87 (p ​< ​0.05).

### 3.6 Performance of multivariable models in predicting HES-kOA

The relationships between DXA-derived variables and HES-kOA reflected those observed with TKR, albeit generally weaker in comparison (Table 4).

As seen for TKR, the model that exhibited the optimal fit and discrimination comprised the osteophyte score, B-score, and demographic variables.

In the ROC analysis, demographic variables alone yielded an AUC of 0.65. However, when the B-score and osteophyte grade were incorporated into the model, the AUC improved to 0.76.

## 4 Discussion

The objective of this study was to assess the feasibility of quantifying knee shape using DXA scans and to investigate the potential prognostic value of a novel imaging biomarker incorporating joint shape for predicting TKR. In addition to capturing conventional features of radiographic kOA, namely JSN (as reflected by mJSW) and osteophytes, an automated model was developed to extract knee shape information using SSM. This information was quantified as a B-score. We found strong correlations between all of the DXA-derived features investigated and subsequent risk of TKR. Similar, although comparatively weaker associations were observed with HES-kOA. Furthermore, combining B-score with osteophyte score and demographic factors resulted in improved accuracy in predicting TKR at 5 years, compared with demographic risk factors alone (AUC ​= ​0.87 compared with 0.73).

Several methodologies have been used to extract additional features from knee images to aid in the diagnosis and prognosis of OA, including the use of Deep Learning approaches, as demonstrated in previous studies on knee radiographs [[23], [24], [25]]. Our study is among the first to examine knee shape using DXA-based SSM. One previous investigation, involving a limited cohort of 109 patients, effectively employed this technique to track changes in knee morphology over 6–12 months [26]. However, in that study, which only focused on the femur and tibia, the authors did not annotate osteophytes separately, limiting the ability to determine if shape contributed additional predictive information. Furthermore, they did not observe overall shape changes such as varus alignment. The association between osteophytes and subsequent TKR in the present analysis is consistent with results obtained in our previous hip DXA investigation [11]. Additionally, a previous study examining hip radiographs demonstrated improved prediction of hip OA through the inclusion of an SSM-derived shape score in conjunction with demographic factors, clinical assessments, and radiologist scores, reinforcing the findings observed in our study [27].

The trend toward varus alignment in individuals with HES-kOA and subsequent TKR in our study is consistent with the recognised varus malalignment characteristic in kOA [4,5]. This alignment may serve as a predisposing factor, focusing weight-bearing forces through the medial compartment, resulting in considerable biomechanical stress. Alternatively, varus may develop due to medial JSN, a well-documented feature of primary kOA. Additionally, we noted an enlargement of the femoral articular surface alongside the varus alignment and reduced mJSW in our analysis. These findings are in keeping with previous research using MRI-based SSM approaches, which report widening and flattening of the femoral condyle in knees affected by osteoarthritis [8,[28], [29], [30]].

By employing a B-score, we were able to investigate whether the relationships we found between knee shape and TKR/HES-kOA were independent of other kOA-related features, namely mJSW and osteophytes. While all three variables showed associations with TKR and HES-kOA in univariable analyses, mJSW was not retained in the most parsimonious model, suggesting mJSW is captured within the SSM-derived B-score. In contrast, both B-score and osteophyte score remained as independent predictors, with their combined effect improving model performance, albeit osteophyte score contributed more significantly. Since our SSM template excluded osteophytes, they are unlikely to have directly contributed to the B-score, suggesting that joint alignment may play a role in the relationship between osteophytes and the progression of kOA. Demographic factors including age, sex, and weight, were also strong predictors of TKR/HES-kOA, underscoring the importance of considering imaging biomarkers in conjunction with these variables rather than substituting them.

Our research adopts a novel approach by developing an imaging biomarker for knee shape using DXA scans. This was made possible through the application of SSM to DXA images obtained from a large sample of individuals within the UKB, in whom follow-up data for THR via HES linkage was available. Given the scale of the study, our SSM could serve as a reference model, facilitating replication and validation. Furthermore, the SSM and the BoneFinder search model will be made publicly available on the BoneFinder website. Another notable strength of our study is that, unlike prior studies using SSM to characterise knee shape, which typically focused on the tibia and femur, our SSM uniquely incorporated the superior patella and fibula. Analysing the four bones together allows for a comprehensive assessment of the joints overall shape, which is important given that the knee joint is a complex biomechanical unit.

Our study has several limitations. Firstly, by excluding participants with previous TKR, we could evaluate the predictive potential of DXA-derived imaging biomarkers for future TKR. However, it is possible that some participants had pre-existing HES-kOA at the time of their DXA scan. It was reassuring to observe broadly consistent relationships between imaging biomarkers and both HES-kOA and TKR. Nonetheless, since our analysis only allowed for cross-sectional examination of HES-kOA relationships, further research is needed to ascertain whether DXA-derived biomarkers can also predict subsequent HES-kOA in individuals with early-stage disease.

Secondly, while knee shape data were obtained from all participants with available DXA scans, osteophyte data, which required manual annotation, were only obtained in a subset. Still, this sample still provided sufficient numbers of individuals with TKR to compare the predictive value of different models. Additionally, It's important to acknowledge that DXA scans were conducted with participants in the supine position, a factor that could impact JSN.

Thirdly, our dataset may have underrepresented TKR cases due to procedures being conducted in the private sector not being captured through HES linkage. Furthermore, the available HES data lacked specification regarding whether diagnoses and procedures applied to the left or right knee. While this could affect effect estimates, it is more likely to reduce effect sizes rather than introduce bias into the study. It is also worth emphasising that surgical decision-making involves a multifaceted consideration of various factors. Therefore, it is possible that some participants may have had advanced disease but were unsuitable for, or chose not to undergo, knee replacement surgery.

Fourthly, there may be issues with generalisability as the UKB population is predominantly white (95%) and has lower rates of all-cause mortality compared to the population at large, reflecting the well-known “healthy volunteer” effect [31]. Moreover, 11% of UKB participants recruited from Scotland and Wales were excluded due to separate systems for HES linkage.

Finally, it is important to stress that our study aimed to investigate the feasibility and effectiveness of using SSM on knee DXA scans to quantify overall knee shape, and to assess the initial predictive capabilities of this approach. Further research, including validation using external datasets, is necessary to confirm the reliability of this approach before clinical implementation and to mitigate the risk of overfitting.

In summary, our study highlights the potential value of SSM as a tool for characterising joint shape in knee DXA scans. Moreover, our findings show that knee shape, when integrated with osteophytes and demographic factors in a predictive model, holds promise for identifying individuals at risk of knee osteoarthritis progression. With lower radiation exposure compared to conventional X-rays, DXA scans could offer a viable alternative for identifying individuals who may benefit from interventions aimed at slowing the progression of the disease. However, further studies in independent cohorts are needed to validate these findings.

## Author contributions

Each author has made significant contributions to the study's conception, design, data acquisition, analysis, and interpretation. Furthermore, all authors helped draft the article before approving the final version of this manuscript.

## Funding

This research was funded in whole, or in part, by the Wellcome Trust [Grant numbers: 209233/Z/17/Z, 223267/Z/21/Z]. BGF is funded by an NIHR Academic Clinical Lectureship. CL was funded by a Sir Henry Dale Fellowship jointly funded by the Wellcome Trust and the Royal Society (223267/Z/21/Z). NCH is supported by grants from Medical Research Council (MRC) [MC_PC_21003; MC_PC_21001] and the NIHR Southampton Biomedical Research Centre. AS was affiliated with the Bristol University at the time of the study conduct and is currently affiliated with Roche Diagnostics International, Clinical Development and Medical Affair. At the time this work was conducted MF was an employee at the University of Bristol. MF is now employed by Boehringer Ingelheim UK & Ireland.

## Conflicts of interest

The other authors have declared no conflicts of interest. For the purpose of open access, the author has applied a CC BY public copyright licence to any Author Accepted Manuscript version arising from this submission.

We confirm that there are no conflicts of interest associated with this manuscript, including any financial support or benefits from commercial sources.
