# Supplementary material for: Dual-energy X-ray absorptiometry derived knee shape may provide a useful imaging biomarker for predicting total knee replacement: Findings from a study of 37,843 people in UK Biobank
Source: Osteoarthr Cartil Open. 2024 Apr 9;6(2):100468. doi: 10.1016/j.ocarto.2024.100468 (PMC11035060; doi:10.1016/j.ocarto.2024.100468)
Supplement: Multimedia component 1 [file mmc1.docx]

**Supplementary Material**

Supplementary Table 1: A list of the ICD-9 and ICD-10 revision codes used for the categorisation of HES-kOA and the OPCS-4 codes for identification of knee replacement.

| **ICD 10 code** | **Condition** |
| --- | --- |
| M17 | Gonarthrosis [arthrosis of knee]. |
| M170 | Primary gonarthrosis, bilateral. |
| M171 | Other primary gonarthrosis. |
| M179 | Gonarthrosis, unspecified. |
| M1906 | Primary arthrosis of other joints, lower leg. |
| M1996 | Arthrosis, unspecified, lower leg. |
| **ICD 9 code** | **Condition** |
| 71536 | Osteoarthrosis, localized, not specified whether primary or secondary, lower leg. |
| 71516 | Localised, primary osteoarthrosis and allied disorders, lower leg. |
| **OPSC-4 code** |  |
| W40 | Total prosthetic replacement of knee joint using cement. |
| W41 | Total prosthetic replacement of knee joint not using cement. |
| W42 | Other total prosthetic replacement of knee joint. |

Cases of hospital diagnosed knee OA (HES-kOA) and total knee replacement (TKR) were identified via linkage to the HES database, which records details of all admissions, outpatient appointments, and A&E attendances at National Health Service (NHS) hospitals in England. The HES database uses the International Classification of Diseases, 9th (ICD-9) and 10th (ICD-10) revision codes to classify diseases and health conditions, while surgical procedures are classified using the Office of Population Censuses and Surveys (OPCS) Classification of Surgical Operations and Procedures, version 4 codes (1). In this study, we obtained the relevant codes from the UKB data fields 41270 (ICD-10), 41271 (ICD-9), and 41273 (OPCS-4), adopting the codes utilized in the study conducted by Zengini et al (2).

Supplementary Table 2: Number of images corrected for point placement during development of the SSM.

| **Bone** | **No. of images with manual point corrections** | **Average point-to-point correction distance (mm)** |
| --- | --- | --- |
| Femur | 812 | 1.8 |
| Tibia | 1,074 | 1.7 |
| Fibula | 2,253 | 4.6 |
| Patella | 1,714 | 4.9 |
| **Total** | **4,214** |  |

The automated search model placed points to 31,207 images. Manual point corrections were made to 4,214 images.

Supplementary Table 3: Creating a DXA-based osteophyte score.

| **Sum of visually graded OPs** | **n** | **OP score** | **n** |
| --- | --- | --- | --- |
| 0 | 3,122 | 0 | 3,122 |
| 1 | 1,757 | 1 | 1,757 |
| 2 | 964 | 2 | 1,393 |
| 3 | 429 |  |  |
| 4 | 169 | 3 | 447 |
| 5 | 95 |  |  |
| 6 | 62 |  |  |
| 7 | 44 |  |  |
| 8 | 32 |  |  |
| 9 | 16 |  |  |
| 10 | 18 |  |  |
| 11 | 6 |  |  |
| 12 | 5 |  |  |

Osteophytes were evaluated in 6,719 DXA images. In the table, the first two columns present the total cumulative sum of manually graded osteophytes, accompanied by the count of individual knees displaying each respective value. Using this cumulative value, an osteophyte score ranging from 0 to 3 was calculated for each knee. The final two columns of the table depict the frequency of knees falling within each of these osteophyte scores.

Supplementary Table 4: Association of top 10 shape modes and B-score with kOA (n=37,843).

|  | **Unadjusted** | | | | **Adjusted** | | | |
| --- | --- | --- | --- | --- | --- | --- | --- | --- |
| TKR | | | | | | | | |
| KSM | HR | lower  95% CI | upper  95% CI | p-value | HR | lower  95% CI | upper  95% CI | p-value |
| 1 | 0.84 | 0.77 | 0.92 | **1.98 x 10^-04^** | 0.92 | 0.84 | 1.01 | 6.82 **x 10**^-02^ |
| 2 | 1.17 | 1.07 | 1.28 | **4.97 x 10^-04^** | 1.10 | 1.01 | 1.21 | 0.034 |
| 3 | 1.01 | 0.92 | 1.10 | 0.894 | 1.03 | 0.94 | 1.14 | 0.512 |
| 4 | 0.88 | 0.80 | 0.96 | 0.005 | 0.87 | 0.80 | 0.96 | **0.004** |
| 5 | 1.08 | 0.99 | 1.19 | 0.080 | 1.05 | 0.96 | 1.15 | 0.269 |
| 6 | 0.85 | 0.77 | 0.93 | **4.24 x 10^-04^** | 0.88 | 0.80 | 0.97 | 0.009 |
| 7 | 1.50 | 1.37 | 1.65 | **7.47 x 10^-18^** | 1.43 | 1.31 | 1.57 | **1.25 x 10^-14^** |
| 8 | 1.71 | 1.56 | 1.87 | **8.69 x 10^-31^** | 1.68 | 1.53 | 1.84 | **8.18 x 10^-29^** |
| 9 | 0.76 | 0.69 | 0.83 | **1.25 x 10^-09^** | 0.83 | 0.75 | 0.91 | **1.29 x 10^-04^** |
| 10 | 0.98 | 0.90 | 1.07 | 0.701 | 0.98 | 0.89 | 1.07 | 0.622 |
| b-score | 2.44 | 2.26 | 2.62 | **<0.001** | 2.25 | 2.08 | 2.43 | **<0.001** |
| HES-kOA | | | | | | | | |
| KSM | OR | lower  95% CI | upper  95% CI | p-value | OR | lower  95% CI | upper  95% CI | p-value |
| 1 | 0.84 | 0.80 | 0.88 | **4.50 x 10^-12^** | 0.91 | 0.87 | 0.96 | **3.00E^-04^** |
| 2 | 1.11 | 1.06 | 1.17 | **2.66 x 10^-05^** | 1.04 | 0.99 | 1.09 | 0.168 |
| 3 | 0.91 | 0.87 | 0.96 | **3.91 x 10^-04^** | 0.97 | 0.92 | 1.03 | 0.332 |
| 4 | 1.00 | 0.95 | 1.05 | 0.944 | 1.00 | 0.95 | 1.05 | 0.899 |
| 5 | 1.02 | 0.97 | 1.07 | 0.357 | 0.99 | 0.94 | 1.04 | 0.742 |
| 6 | 0.93 | 0.88 | 0.97 | **0.003** | 0.96 | 0.91 | 1.01 | 0.087 |
| 7 | 1.32 | 1.25 | 1.39 | **2.83 x 10^-27^** | 1.28 | 1.22 | 1.35 | **9.05 x 10^-22^** |
| 8 | 1.37 | 1.30 | 1.44 | **3.85 x 10^-35^** | 1.33 | 1.26 | 1.40 | **5.29 x 10^-28^** |
| 9 | 0.86 | 0.82 | 0.91 | **3.00 x 10^-09^** | 0.90 | 0.86 | 0.95 | **2.01 x 10^-04^** |
| 10 | 0.95 | 0.90 | 0.99 | 0.026 | 0.92 | 0.87 | 0.97 | **0.001** |
| b-score | 1.94 | 1.85 | 2.03 | **<0.001** | 1.80 | 1.71 | 1.89 | **<0.001** |

Hazard ratios (HRs) and odds ratios (ORs) represent the change in risk of total knee replacement (TKR) and hospital diagnosed knee osteoarthritis (HES-kOA) per standard deviation increase in knee shape mode (KSM). Models were adjusted for age, sex, height and weight. Associations that met the Bonferroni-significant threshold of *P*<0.005 are shown in bold. CI, 95% confidence intervals.

Supplementary Table 5: variation described by the top 10 knee shape modes (KSMs).

| Mode  (% of variation) | Shape +2SD | Changes Associated with a -2SD decrease |
| --- | --- | --- |
| 1 (22.9%) | 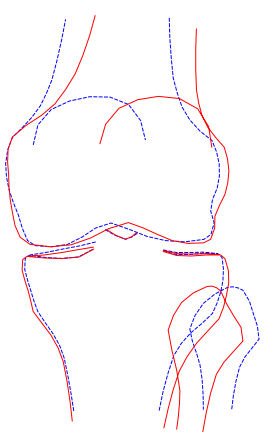 | - Varus alignment of the femur. - Medial patellar displacement. - Lateral shift of the fibula. |
| 2 (15.3%) | 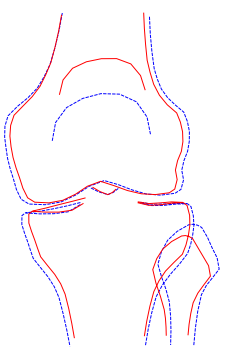 | - Lower patella height. - Widening of the Femur and Tibia. - Taller fibula head. |
| 3 (11.7%) | 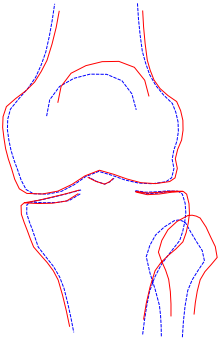 | - Lower patella height, accompanied by a medial shift. - Medial Displacement and Lowered Position of the Fibula. - Reduction in the width of the tibial plateau. - Reduction in the width of the femoral plateau and condyles. |
| 4 (7.9%) | 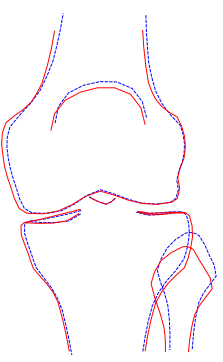 | - Elevated Position of the Fibula. - Narrowing of the Medial Femoral Width. - Slight Elevation in Patella Height. |
| 5 (5.0%) | 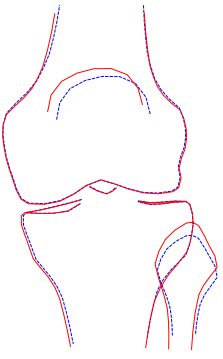 | - Moderate lateral shift and lower positioning of the patella - Downward positioning of the fibula. - Reduced depth of the fibula head. |
| 6 (4.3%) | 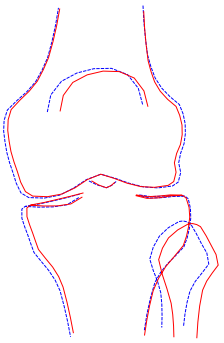 | - Moderate medial displacement of the patella. - Medial shift of the fibula. - Widening of the medial tibia. - Widening in the proximal region of the femur. |
| 7 (4.0%) | 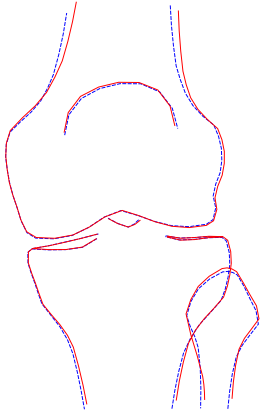 | - Medial shift in the femoral shaft. |
| 8 (3.4%) | 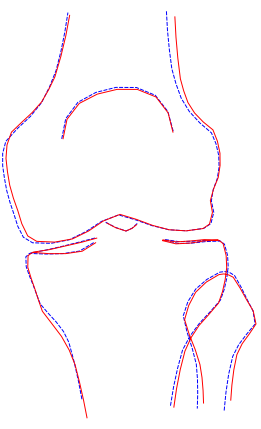 | - Narrowing of the femoral shaft, laterally. - Increased curvature beneath the medial tibial condyle. |
| 9 (3.2%) | 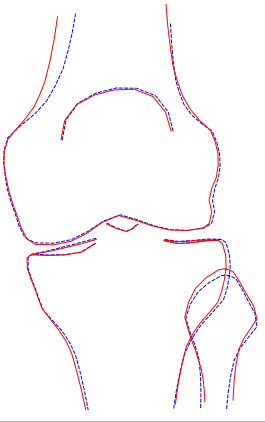 | - Narrowing of the medial femoral shaft. - Smaller fibular head. |
| 10 (2.6%) | 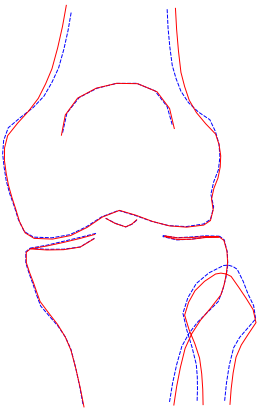 | - Narrowing of the femoral shaft. - Larger fibular head. |

+ 2SD

- 2SD

The first column in the table displays the extent of shape variation captured by each statistical knee shape mode (KSM). In the second column, visual representations of the mode are presented, depicting solid red lines for shapes corresponding to values 2 standard deviations (SD) above the mean mode shape, and dashed blue lines for shapes associated with values 2 SD below the mean mode shape. The last column offers a description of the alterations related to a 2 SD decrease in KSM.

Supplementary Table 6: Association of sex-specific and sex-combined B-scores with kOA outcomes (n=37,843).

| Unadjusted | | | | | Adjusted | | | | | |
| --- | --- | --- | --- | --- | --- | --- | --- | --- | --- | --- |
| **TKR** | | | | | | | | | | |
| **B-score** | N | HR | lower  95% CI | upper 95% CI | p-value |  | HR | lower  95% CI | upper 95% CI | p-value |
| Male | 18133 | 2.55 | 2.30 | 2.82 | <0.001 |  | 2.38 | 2.15 | 2.65 | <0.001 |
| Female | 19710 | 2.47 | 2.21 | 2.75 | <0.001 |  | 2.21 | 1.97 | 2.48 | <0.001 |
| **HES-kOA** | | | | | | | | | | |
| **B-score** | N | OR | lower  95% CI | upper 95% CI | p-value |  | OR | lower  95% CI | upper 95% CI | p-value |
| Male | 18133 | 1.92 | 1.80 | 2.04 | <0.001 |  | 1.82 | 1.70 | 1.94 | <0.001 |
| Female | 19710 | 1.99 | 1.86 | 2.13 | <0.001 |  | 1.81 | 1.68 | 1.94 | <0.001 |

Hazard ratios (HRs) and Odds ratios (ORs) reflect the change in risk for total knee replacement (TKR) (top panel) and hospital diagnosed knee osteoarthritis (HES-kOA) (bottom panel) per standard deviation increase in B-score. Adjusted models incorporate age, sex, height, and weight as covariates. CI, 95% confidence intervals.  *p-*values akin to 0.00E+00.

Supplementary Table 7: Unadjusted associations of minimum joint space width (mJSW) with HES-kOA and TKR (n=37,843).

|  | TKR | | | |  | HES-kHOA | | | |
| --- | --- | --- | --- | --- | --- | --- | --- | --- | --- |
|  | *HR* | *ll* | *ul* | *p-value* |  | *OR* | *ll* | *ul* | *p-value* |
| *Medial compartment* |  |  |  |  |  |  |  |  |  |
| 1st quartile | 2.60 | 2.01 | 3.36 | **2.22 x 10^-13^** |  | 1.40 | 1.22 | 1.60 | **9.42 x 10^-07^** |
| 2nd quartile | 1.27 | 0.95 | 1.70 | 0.105 |  | 1.00 | 0.86 | 1.15 | 0.970 |
| 3rd quartile | 0.95 | 0.70 | 1.30 | 0.756 |  | 0.91 | 0.79 | 1.06 | 0.231 |
| *Lateral compartment* |  |  |  |  |  |  |  |  |  |
| 1st quartile | 0.81 | 0.63 | 1.03 | 0.083 |  | 0.73 | 0.64 | 0.83 | **3.92 x 10^-06^** |
| 2nd quartile | 0.62 | 0.48 | 0.80 | **3.21 x 10^-04^** |  | 0.66 | 0.57 | 0.75 | **2.36 x 10^-09^** |
| 3rd quartile | 0.82 | 0.65 | 1.04 | 0.104 |  | 0.79 | 0.69 | 0.90 | **3.88 x 10^-04^** |
| *Binary Q1 vs >Q1* | 2.42 | 2.02 | 2.90 | **<0.001** |  | 1.44 | 1.30 | 1.60 | 1.25 **x 10**^-11^ |

When examining the association between quartiles of minimum joint space width (mJSW) and the risk of total knee replacement (TKR) and hospital diagnosed knee osteoarthritis (HES-kOA), we designated quartile 4 as the reference category (i.e., the greatest mJSW). Odds ratios (ORs) and hazards ratios (HRs) represent the difference in risk for someone in the first, second or third quartile versus the fourth quartile. For the binary mJSW variable, the HRs and ORs quantify the risk variation associated with values above the first quartile in the medial compartment, in comparison with the first quartile (representing the “unhealthiest” mJSW). CI, 95% confidence interval; Q1, first quartile of mJSW (medial compartment). p<0.05 are shown in bold.

Supplementary Table 8: Prevalence of manually graded osteophytes by site (n=6,719).

|  | **Medial femur** | |  | **Lateral femur** | |  | **Medial tibia** | |  | **Lateral tibia** | |
| --- | --- | --- | --- | --- | --- | --- | --- | --- | --- | --- | --- |
| *Grade* | *N* | *%* |  | *N* | *%* |  | *N* | *%* |  | *N* | *%* |
| 0 | 4812 | 71.62 |  | 6218 | 92.54 |  | 5126 | 76.29 |  | 4399 | 65.47 |
| 1 | 1538 | 22.89 |  | 292 | 4.35 |  | 1414 | 21.04 |  | 2136 | 31.79 |
| 2 | 264 | 3.93 |  | 133 | 1.98 |  | 150 | 2.23 |  | 147 | 2.19 |
| 3 | 105 | 1.56 |  | 76 | 1.13 |  | 29 | 0.43 |  | 37 | 0.55 |

Supplementary Table 9: Results of the unadjusted regression analysis examining the association of osteophyte grades with TKR and HES-kOA (n=6,719).

|  | Unadjusted | | | | | | | | |
| --- | --- | --- | --- | --- | --- | --- | --- | --- | --- |
|  | **TKR** | | | |  | **HES-kOA** | | | |
|  | *HR* | *ll* | *ul* | *p-value* |  | *OR* | *ll* | *ul* | *p-value* |
| **Medial femur** | |  |  |  |  |  |  |  |  |
| grade 1 | 2.82 | 1.86 | 4.29 | 1.21 x 10^-06^ |  | 1.65 | 1.28 | 2.11 | 8.24 x 10^-05^ |
| grade 2 | 13.73 | 8.76 | 21.52 | 3.36 x 10^-30^ |  | 7.58 | 5.52 | 10.39 | 3.24 x 10^-36^ |
| grade 3 | 30.69 | 19.12 | 49.28 | <0.001 |  | 11.84 | 7.70 | 18.20 | 2.15 x 10^-29^ |
| **Lateral femur** | |  |  |  |  |  |  |  |  |
| grade 1 | 2.89 | 1.62 | 5.16 | 3.25 x 10^-04^ |  | 2.41 | 1.64 | 3.54 | 7.44 x 10^-06^ |
| grade 2 | 9.62 | 5.82 | 15.92 | 1.22 x 10^-18^ |  | 7.84 | 5.29 | 11.61 | 1.16 x 10^-24^ |
| grade 3 | 19.67 | 12.15 | 31.86 | 9.21 x 10-34 |  | 7.98 | 4.80 | 13.28 | 1.27 x 10^-15^ |
| **Medial tibia** | |  |  |  |  |  |  |  |  |
| grade 1 | 4.47 | 3.08 | 6.49 | 2.96 x 10^-15^ |  | 3.52 | 2.82 | 4.39 | 3.67 x 10^-29^ |
| grade 2 | 18.51 | 11.47 | 29.87 | 6.54 x 10^-33^ |  | 8.22 | 5.48 | 12.34 | 2.78 x 10^-24^ |
| grade 3 | 44.55 | 22.61 | 87.76 | 5.05 x 10^-28^ |  | 21.95 | 10.40 | 46.30 | 5.12 x 10^-16^ |
| **Lateral tibia** | |  |  |  |  |  |  |  |  |
| grade 1 | 3.60 | 2.47 | 5.25 | 2.72 x 10^-11^ |  | 2.30 | 1.85 | 2.86 | 5.15 x 10^-14^ |
| grade 2 | 13.69 | 7.90 | 23.74 | 1.17 x 10^-20^ |  | 7.01 | 4.60 | 10.68 | 1.25 x 10^-19^ |
| grade 3 | 46.44 | 24.97 | 86.40 | 8.29 x 10^-34^ |  | 17.17 | 8.75 | 33.70 | 1.38 x 10^-16^ |
| **OP Score** | 8.59 | 5.90 | 12.51 | 3.66 x 10^-29^ |  | 4.11 | 3.34 | 5.06 | <0.001 |
| 1 | 2.02 | 1.05 | 3.88 | 0.036 |  | 1.68 | 1.23 | 2.30 | 0.001 |
| 2 | 5.73 | 3.26 | 10.07 | 1.24 x 10^-09^ |  | 3.18 | 2.39 | 4.25 | 3.47 x 10^-15^ |
| 3 | 32.05 | 18.85 | 54.49 | 1.65 x 10^-37^ |  | 12.52 | 9.26 | 16.94 | <0.001 |

The reference category for the regressions was set as grade 0. Abbreviations: CI, 95% confidence interval; HR, hazard ratio; OR, odds ratio.

Supplementary Figure 1: An example DXA image with the 129-point SSM template applied.


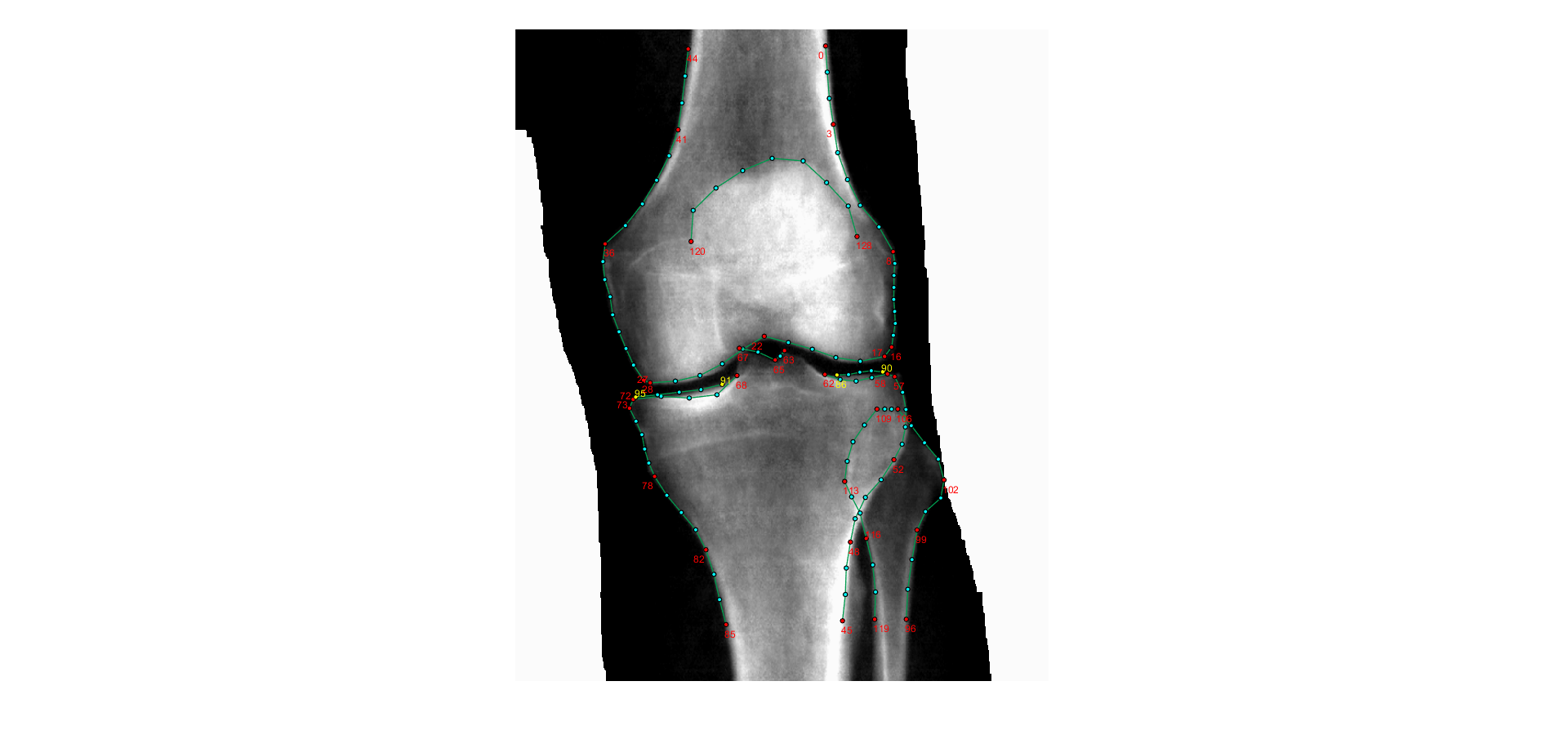


The image captured by the iDXA scanner displays the outline of a left knee and is overlapped by the statistical shape model (SSM) template. Landmark points, indicated in red and yellow (anterior tibial plateau), are placed on distinctive anatomical features and the remaining points are evenly distributed between each pair of landmarks in order to describe the shape.

Supplementary Figure 2: Results of the 3-fold cross-validation experiment used to evaluate the automated search model.


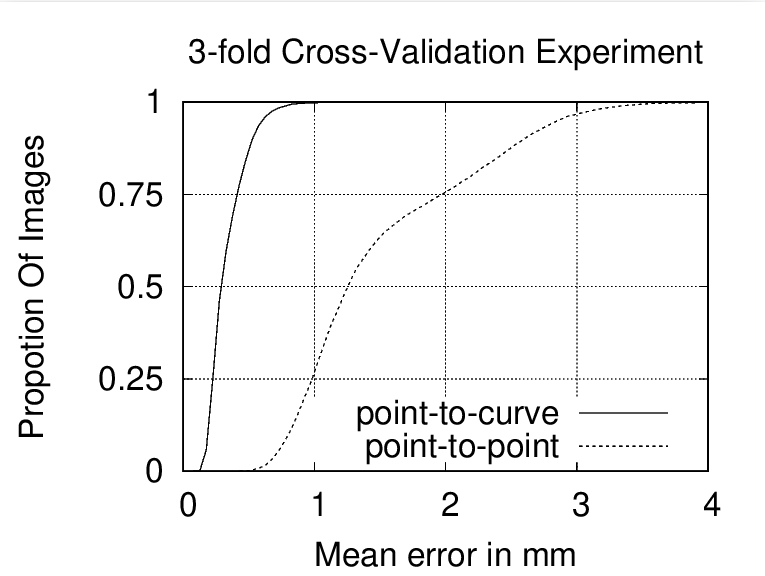


The model was evaluated using the Euclidean distance between the automatic points and the manual points (i.e., point-to-point error), and the curve passing through manual points (i.e., point-to-curve error). Mean point-to-point error, and point-to-curve error were under 3mm, and 0.7 mm respectively in 95% of the images.

Supplementary Figure 3: A graphical representation of the variance explained by each statistical shape model mode.

The proportion of variance explained by each mode is displayed on the y-axis and the number of modes of variation on the x-axis. The blue line shows the decreasing variance explained by individual modes, while the orange line displays the cumulative variance explained as additional modes are included. We used the first ten modes as these each contain more than 2% of the variance.

Supplementary Figure 4: Distribution of B-scores of healthy and diseased examples (n=37,843).


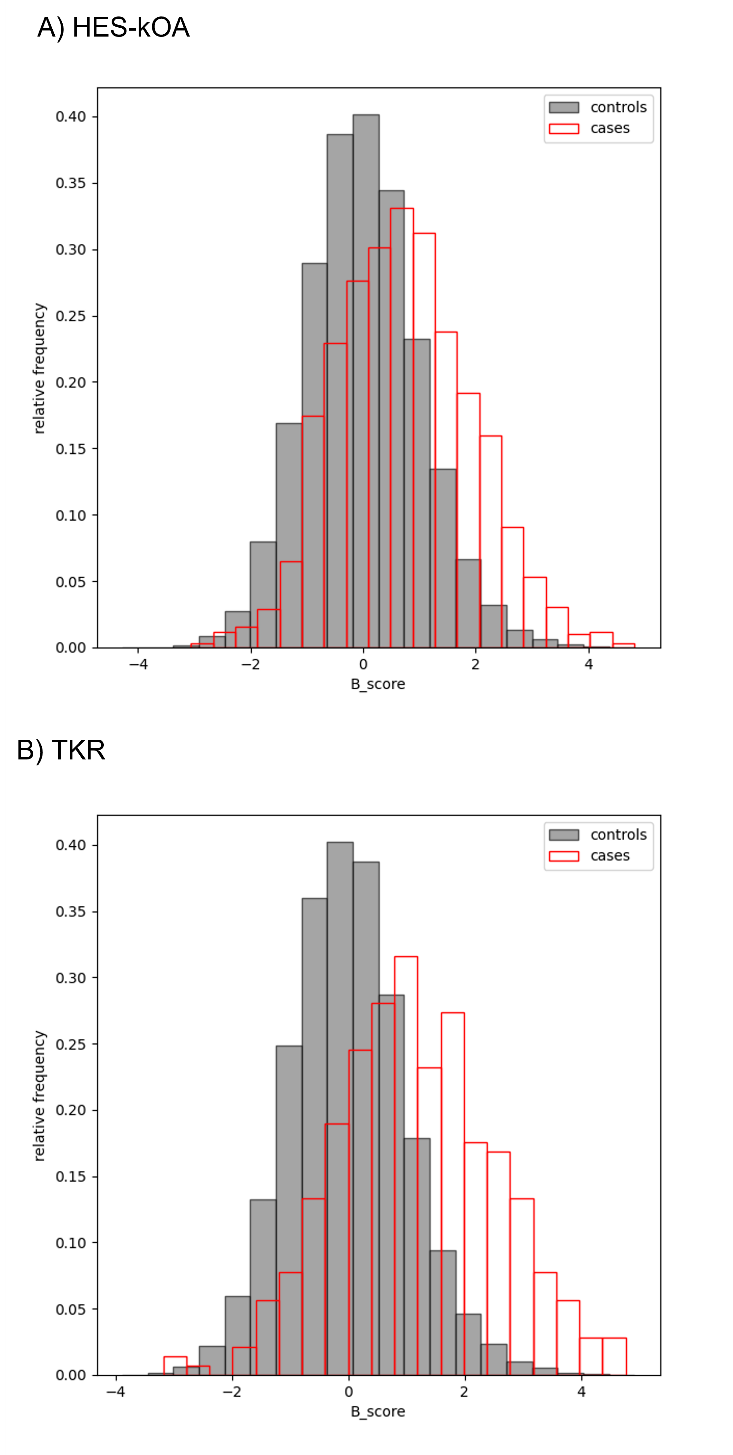


The plots depict the relative frequency of B-scores for healthy individuals in comparison to: A) hospital knee osteoarthritis (HES-kOA), and B) total knee replacement (TKR).

**References**

1. UK Biobank, [Internet]. Hospital inpatient data Version 3; 2020 [cited 04.04.2023]. Available from: <https://biobank.ndph.ox.ac.uk/showcase/showcase/docs/HospitalEpisodeStatistics.pdf>.

2. Zengini E, Hatzikotoulas K, Tachmazidou I, Steinberg J, Hartwig FP, Southam L, et al. Genome-wide analyses using UK Biobank data provide insights into the genetic architecture of osteoarthritis. Nat Genet. 2018;50(4):549-58.

3. Cootes TF, Hill A, Taylor CJ, Haslam J. Use of active shape models for locating structures in medical images. Image and Vision Computing. 1994;12(6):355-65.

4. Gregory JS, Testi D, Stewart A, Undrill PE, Reid DM, Aspden RM. A method for assessment of the shape of the proximal femur and its relationship to osteoporotic hip fracture. Osteoporos Int. 2004;15(1):5-11.

5. Frysz M, Faber BG, Ebsim R, Saunders FR, Lindner C, Gregory JS, et al. Machine Learning-Derived Acetabular Dysplasia and Cam Morphology Are Features of Severe Hip Osteoarthritis: Findings From UK Biobank. J Bone Miner Res. 2022;37(9):1720-32.

6. Bowes MA, Kacena K, Alabas OA, Brett AD, Dube B, Bodick N, et al. Machine-learning, MRI bone shape and important clinical outcomes in osteoarthritis: data from the Osteoarthritis Initiative. Ann Rheum Dis. 2021;80(4):502-8.

7. Gregory JS, Barr RJ, Yoshida K, Alesci S, Reid DM, Aspden RM. Statistical shape modelling provides a responsive measure of morphological change in knee osteoarthritis over 12 months. Rheumatology (Oxford). 2020;59(9):2419-26.

8. Lindner C, Thiagarajah S, Wilkinson JM, Wallis GA, Cootes TF, arc OC. Accurate bone segmentation in 2D radiographs using fully automatic shape model matching based on regression-voting. Med Image Comput Comput Assist Interv. 2013;16(Pt 2):181-9.

9. Haverkamp DJ, Schiphof D, Bierma-Zeinstra SM, Weinans H, Waarsing JH. Variation in joint shape of osteoarthritic knees. Arthritis Rheum. 2011;63(11):3401-7.

10. Minciullo L, Parkes MJ, Felson DT, Cootes TF. Comparing image analysis approaches versus expert readers: the relation of knee radiograph features to knee pain. Ann Rheum Dis. 2018;77(11):1606-9.

11. BoneFinder; 2015 [cited 09.08.2023]. Available from: <https://bone-finder.com/>.

12. Lane NE, Nevitt MC, Hochberg MC, Hung YY, Palermo L. Progression of radiographic hip osteoarthritis over eight years in a community sample of elderly white women. Arthritis Rheum. 2004;50(5):1477-86.

13. Faber BG, Ebsim R, Saunders FR, Frysz M, Lindner C, Gregory JS, et al. Osteophyte size and location on hip DXA scans are associated with hip pain: Findings from a cross sectional study in UK Biobank. Bone. 2021;153:116146.

14. Felson DT, Gale DR, Elon Gale M, Niu J, Hunter DJ, Goggins J, et al. Osteophytes and progression of knee osteoarthritis. Rheumatology (Oxford). 2005;44(1):100-4.
