# Supplementary material for: Dual-energy X-ray absorptiometry derived knee shape may provide a useful imaging biomarker for predicting total knee replacement: Findings from a study of 37,843 people in UK Biobank
Source: Osteoarthr Cartil Open. 2024 Apr 9;6(2):100468. doi: 10.1016/j.ocarto.2024.100468 (PMC11035060; doi:10.1016/j.ocarto.2024.100468)
Supplement: Multimedia component 2 [file mmc2.pdf]

Knee Osteophyte Atlas

Background:

This atlas provided serves as a reference for assessing osteophytes in knee DXA scans. During its development, we consulted the current 'gold standard' system for grading radiographic features of osteoarthritis in clinical trials, as devised by Altman & Gold: https://www.oarsijournal.com/article/S1063-4584(06)00328-1/fulltext. Our DXA atlas was formulated using a subset of 6,718 DXA scans selected for the initial search model. For every anatomical site, we present a representative example sourced from the radiographic atlas, followed by a corresponding illustration extracted from the DXA scans.

We would like to thank Dr. David Wilson, Consultant Interventional Musculoskeletal Radiologist and honorary Clinical Lecturer at Aberdeen University, for his valuable contribution to the development of the DXA-based osteophyte Atlas.

Medial Femoral osteophyte (X-ray)

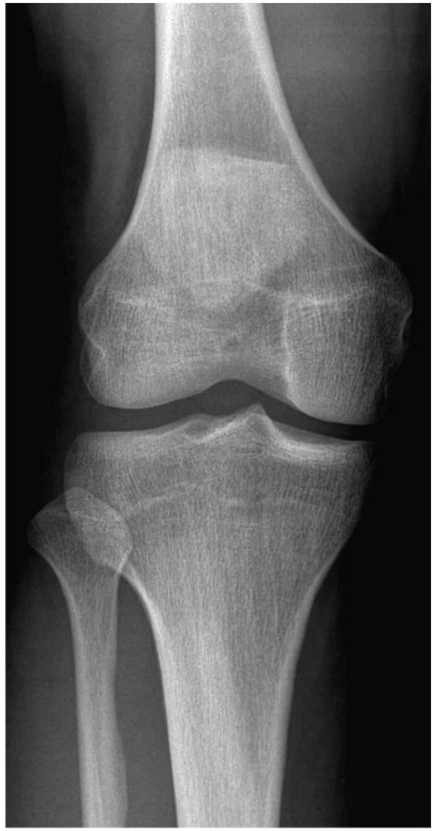

Grade 0

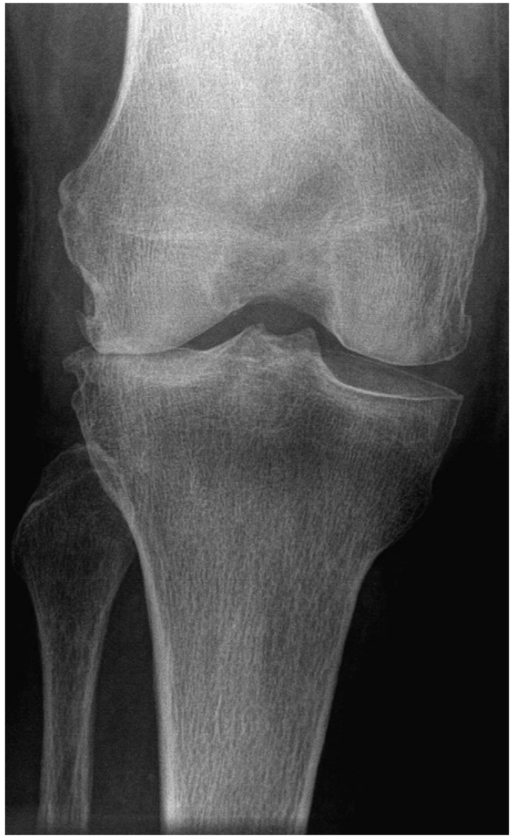

Grade 1

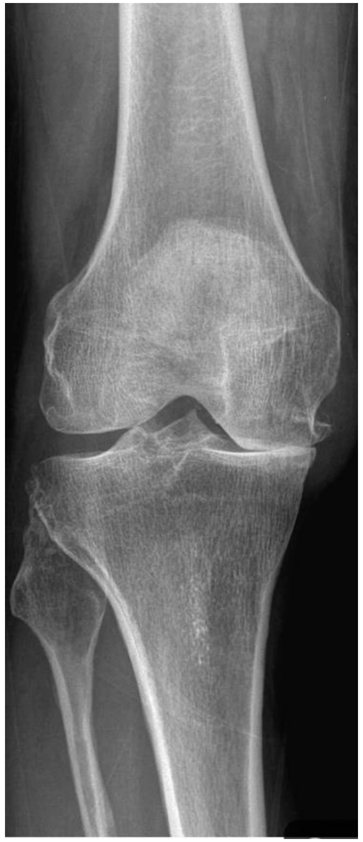

Grade 2

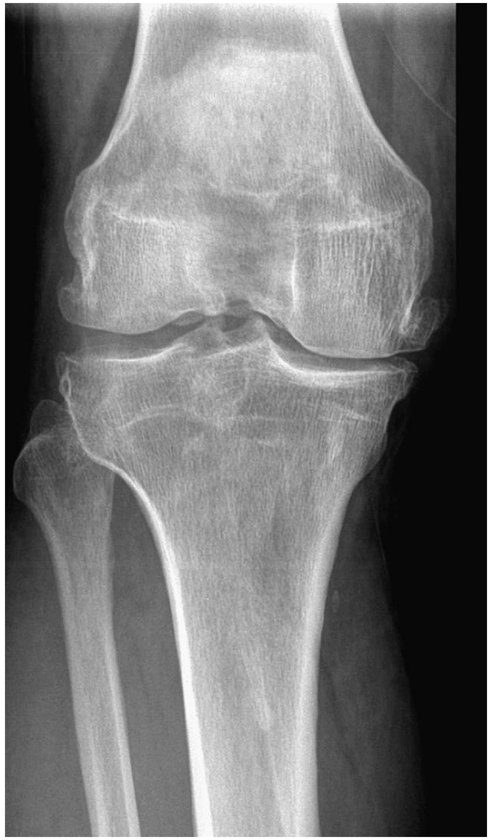

Grade 3

Medial femoral osteophyte (DXA)

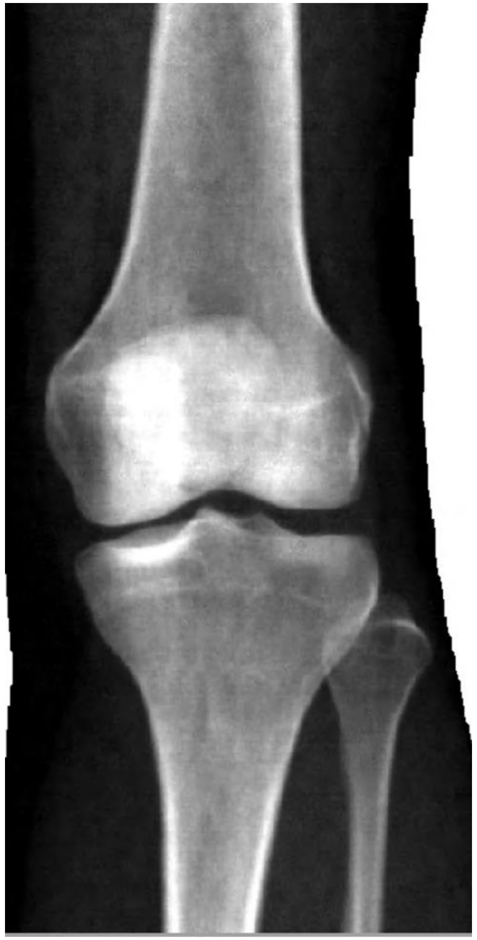

Grade 0

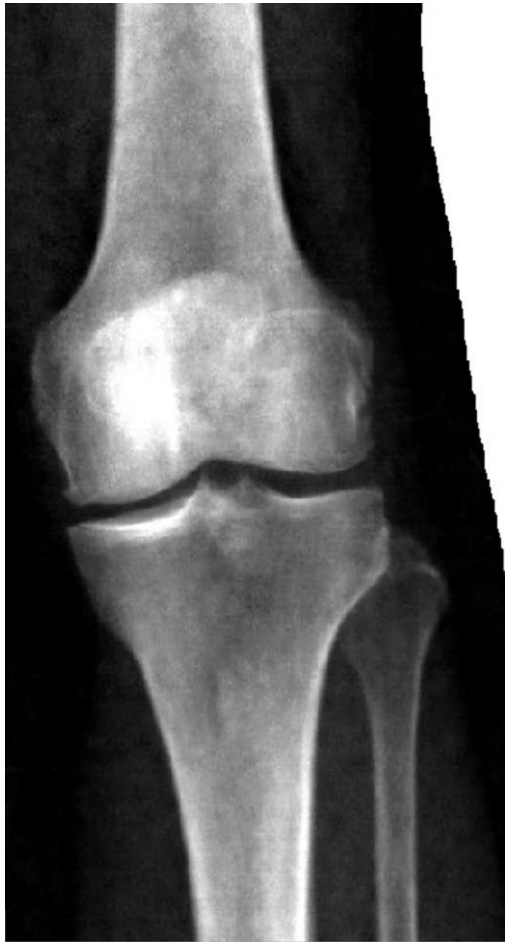

Grade 1

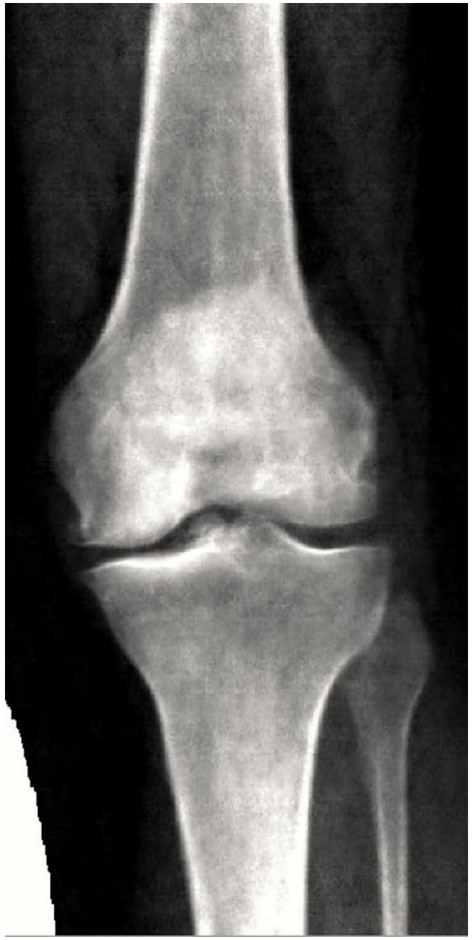

Grade 2

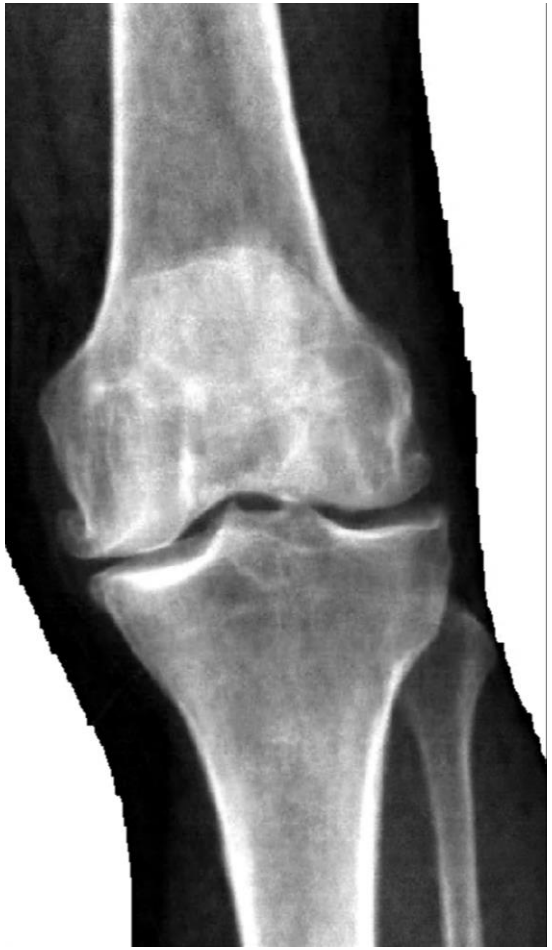

Grade 3

lateral Femoral osteophyte (X-ray)

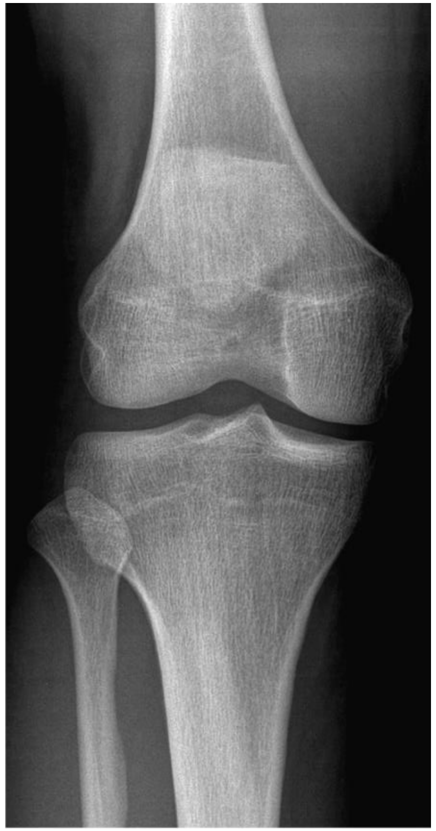

Grade 0

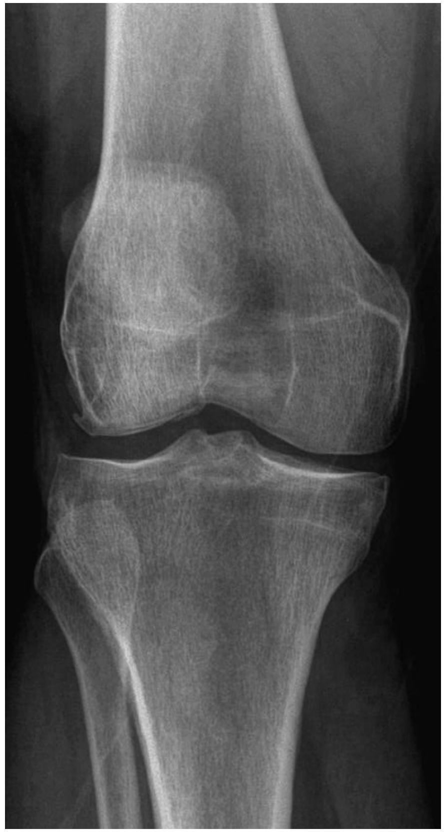

Grade 1

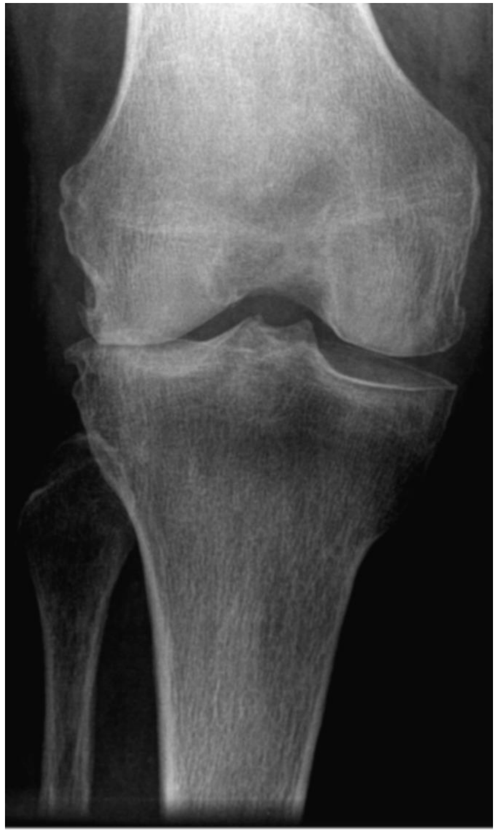

Grade 2

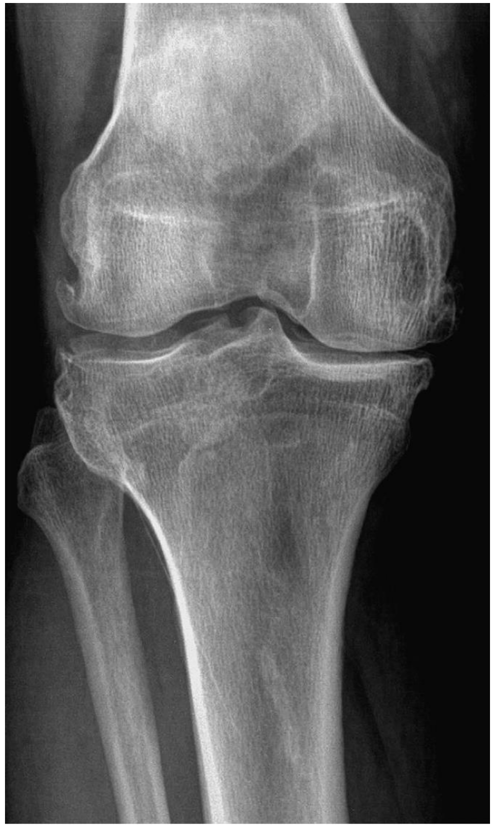

Grade 3

Lateral femoral osteophyte (DXA)

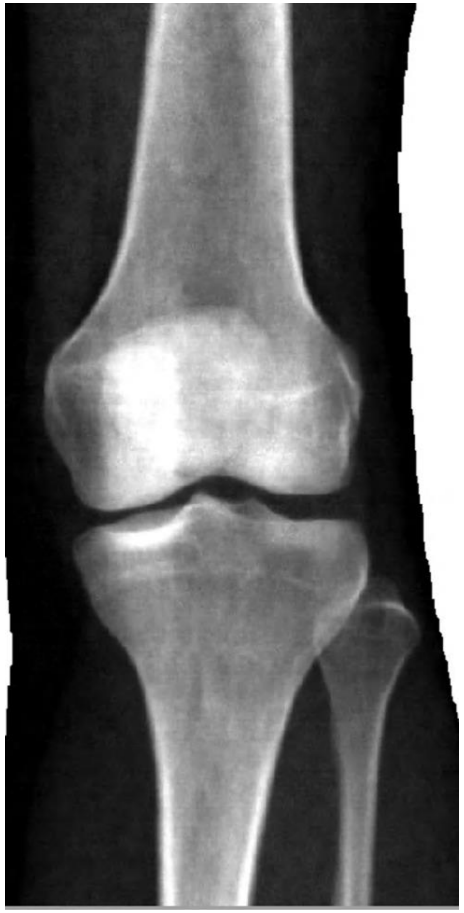

Grade 0

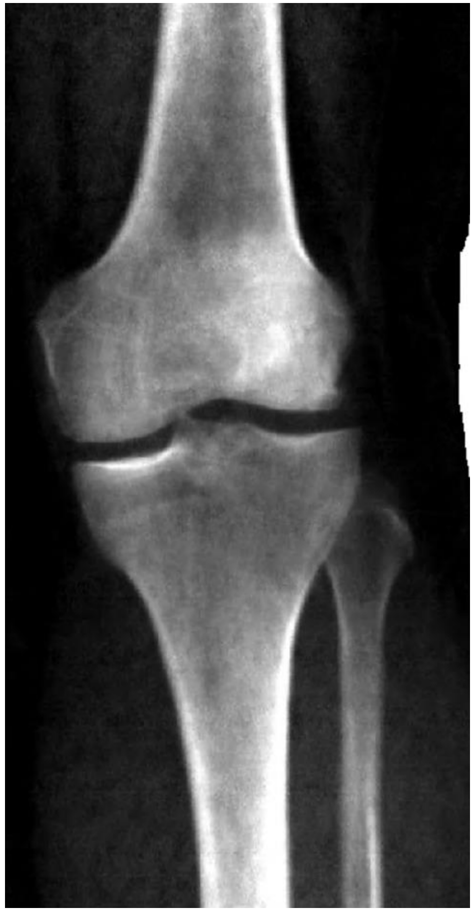

Grade 1

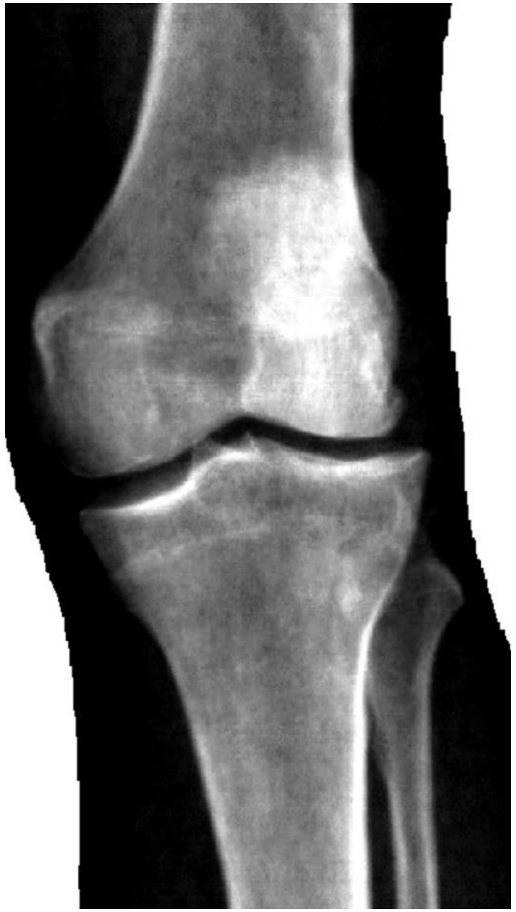

Grade 2

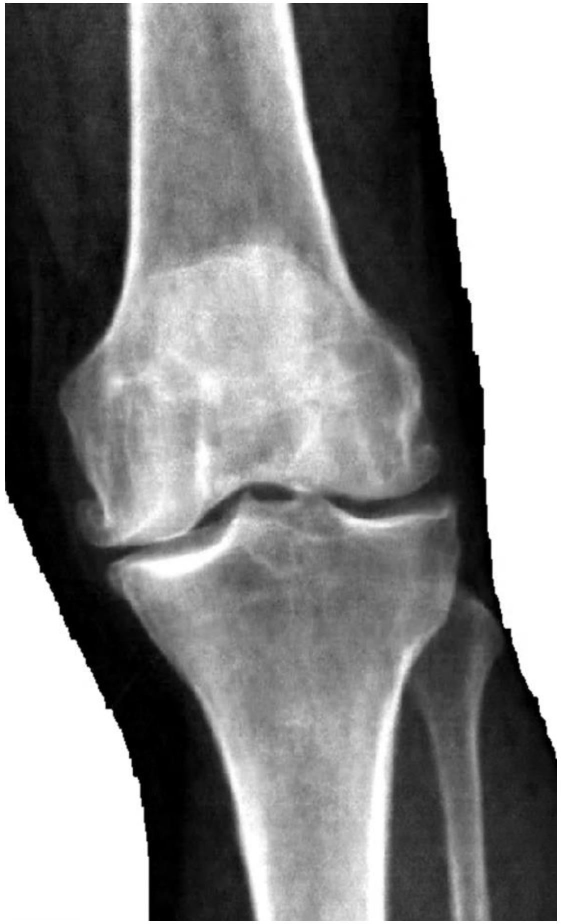

Grade 3

Medial tibial osteophyte (X-ray)

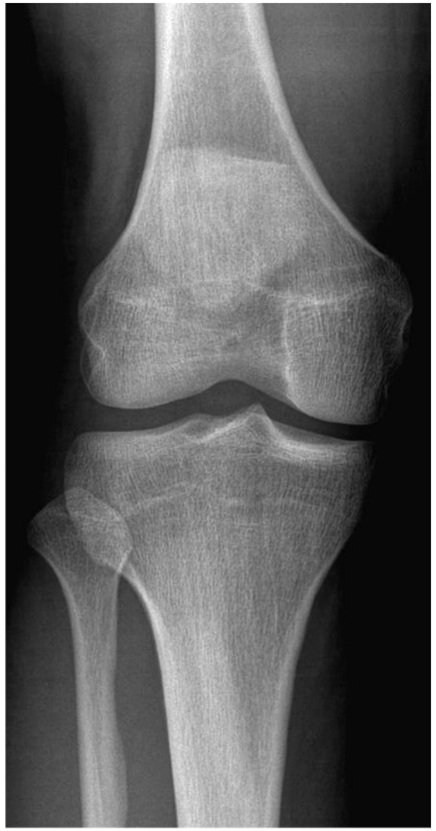

Grade 0

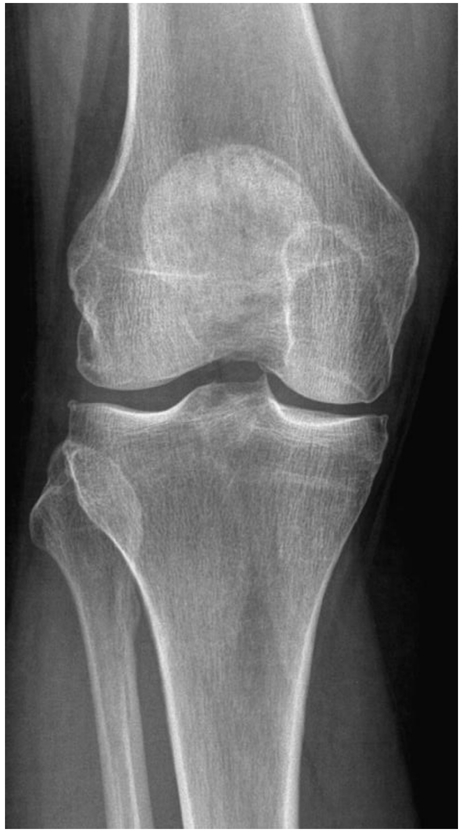

Grade 1

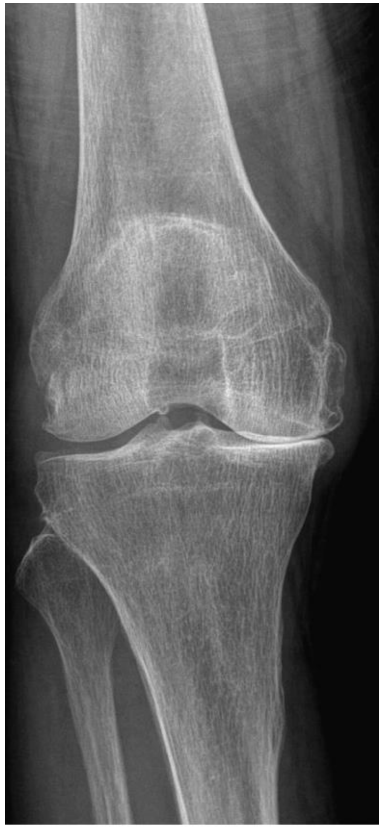

Grade 2

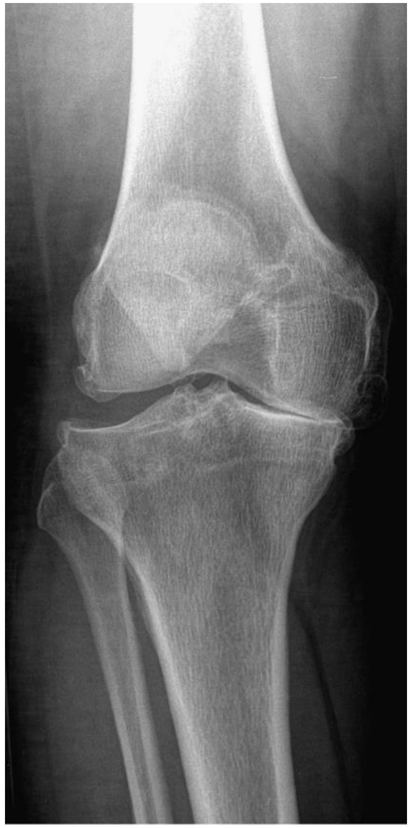

Grade 3

Medial tibial osteophyte (DXA)

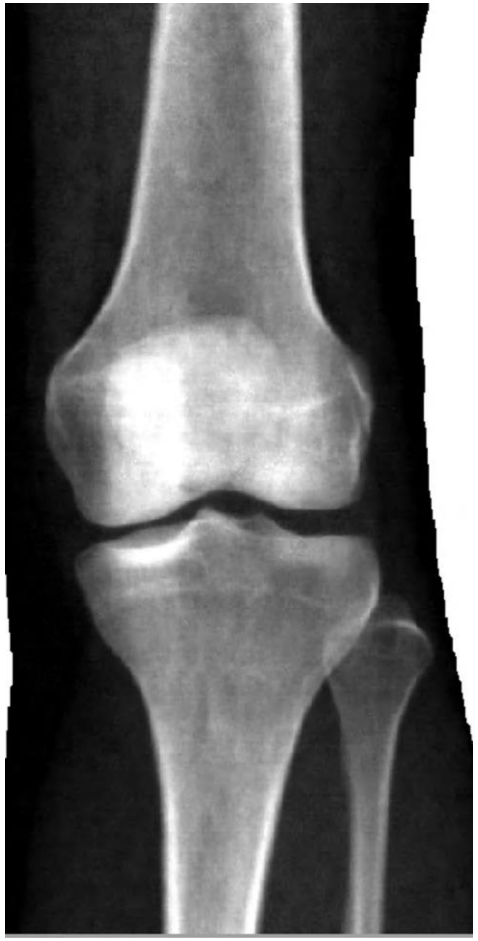

Grade 0

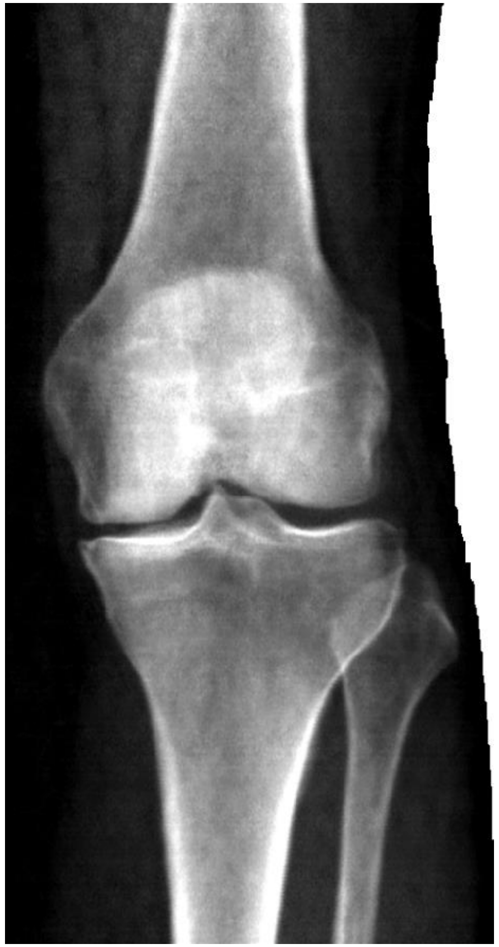

Grade 1

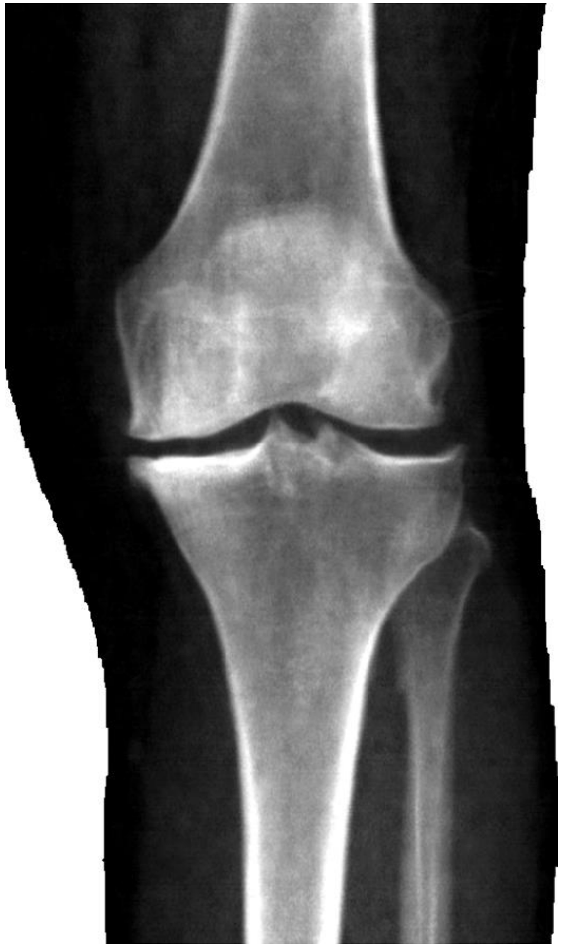

Grade 2

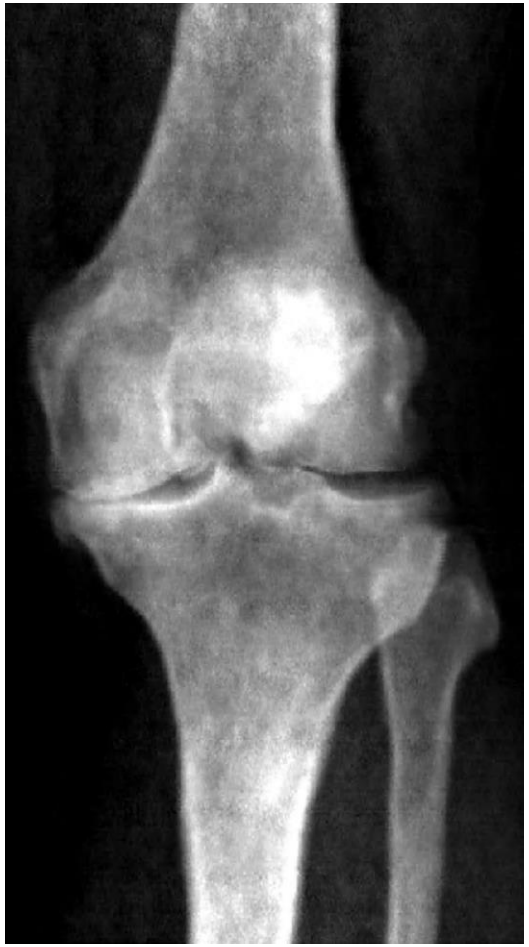

Grade 3

Lateral tibial osteophyte (X-ray)

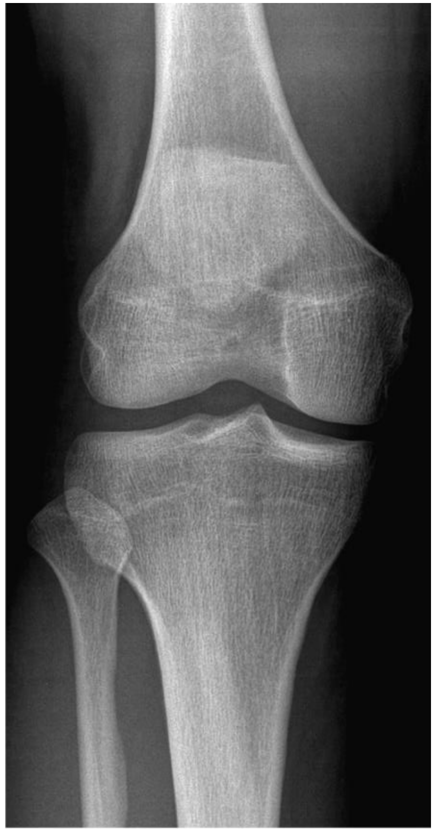

Grade 0

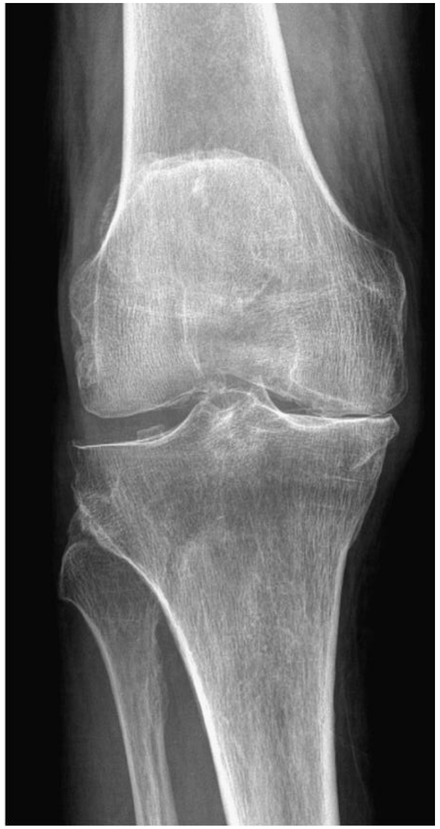

Grade 1

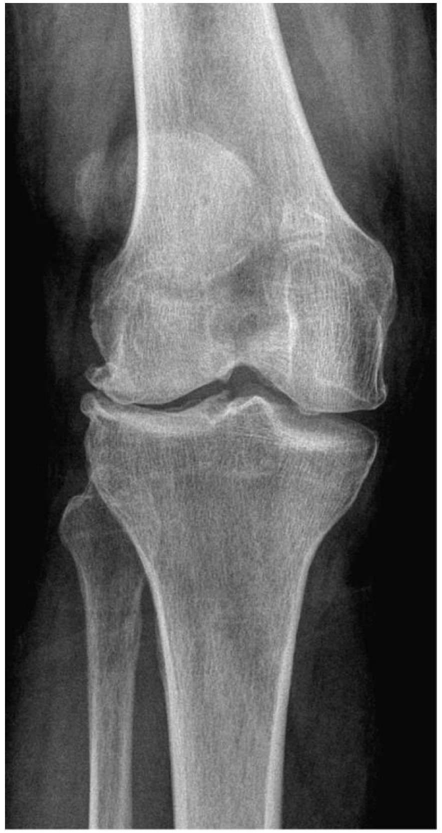

Grade 2

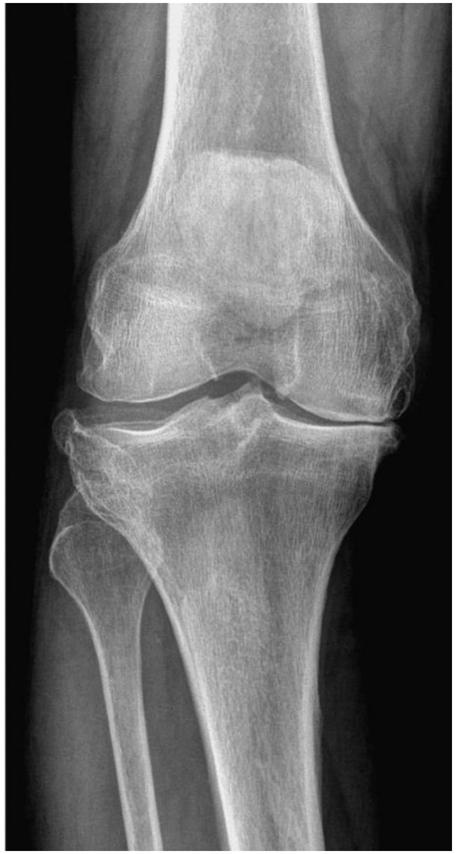

Grade 3

Lateral tibial osteophyte (DXA)

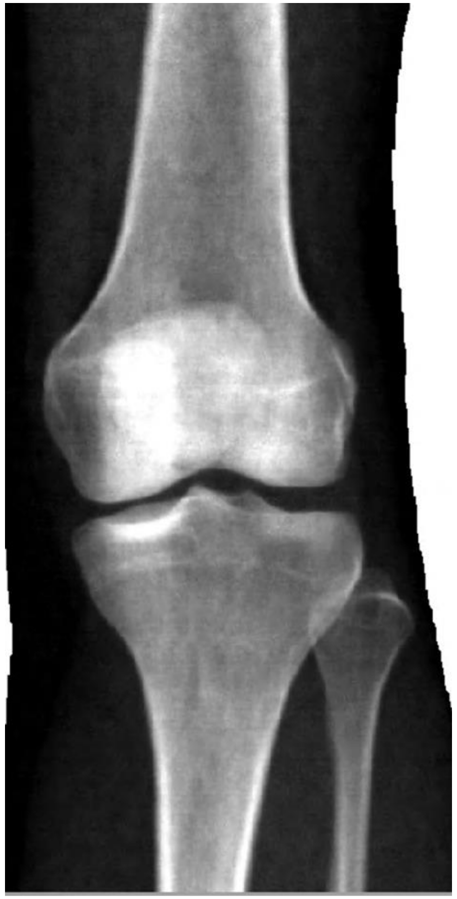

Grade 0

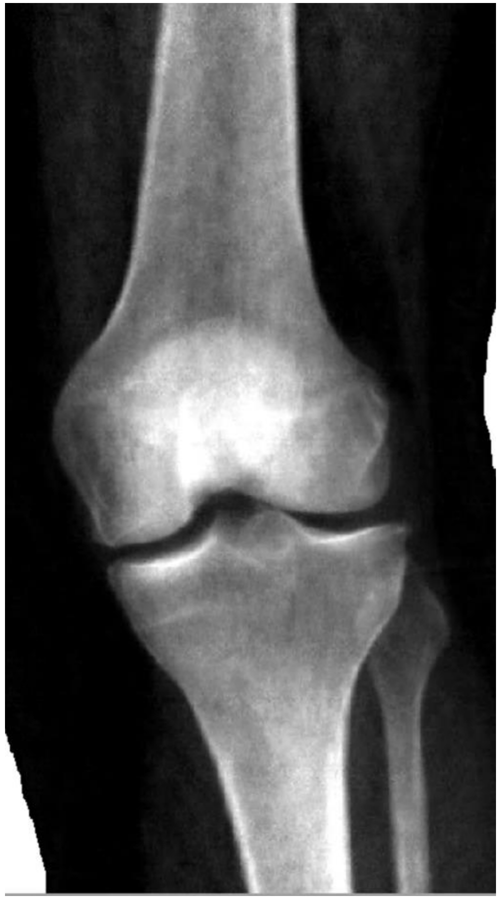

Grade 1

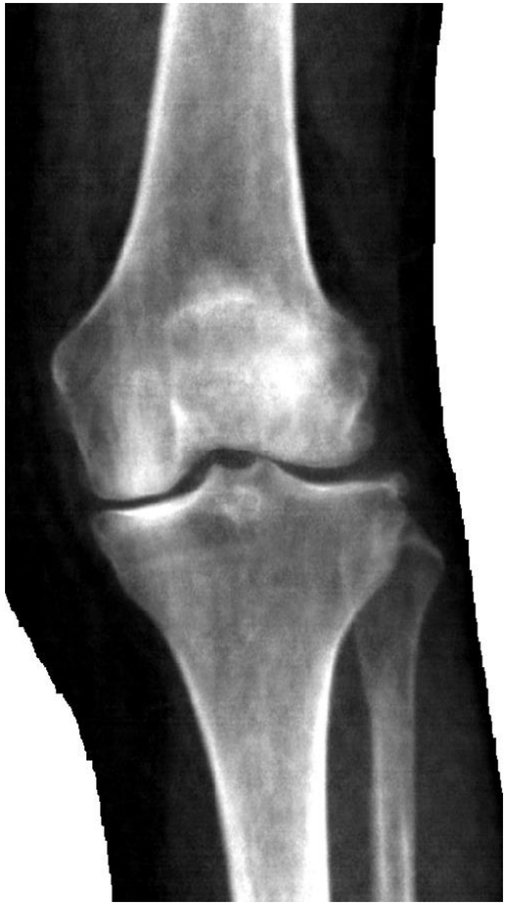

Grade 2

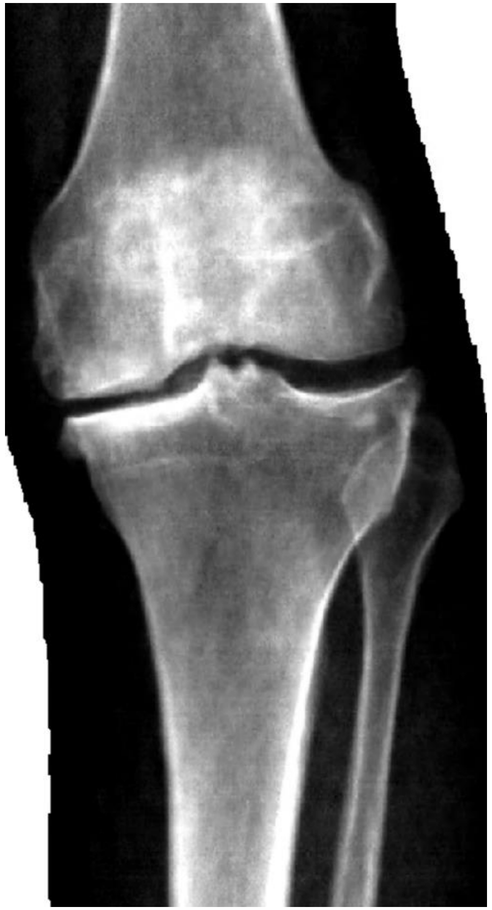

Grade 3
